# Characterizing major depressive disorder and substance use disorder using heatmaps and variable interactions: The utility of operant behavior and brain structure relationships

**DOI:** 10.1371/journal.pone.0299528

**Published:** 2024-03-11

**Authors:** Nicole L. Vike, Sumra Bari, Byoung Woo Kim, Aggelos K. Katsaggelos, Anne J. Blood, Hans C. Breiter, on behalf of the Massachusetts General Hospital Phenotype Genotype Project

**Affiliations:** 1 Department of Computer Science, University of Cincinnati, Cincinnati, Ohio, United States of America; 2 Department of Electrical and Computer Engineering, Northwestern University, Evanston, Illinois, United States of America; 3 Department of Computer Science, Northwestern University, Evanston, Illinois, United States of America; 4 Department of Radiology, Northwestern University, Chicago, Illinois, United States of America; 5 Department of Psychiatry, Mood and Motor Control Laboratory (MAML), Massachusetts General Hospital and Harvard Medical School, Boston, Massachusetts, United States of America; 6 Department of Psychiatry, Laboratory of Neuroimaging and Genetics, Massachusetts General Hospital and Harvard School of Medicine, Boston, Massachusetts, United States of America; 7 Department of Biomedical Engineering, University of Cincinnati, Cincinnati, Ohio, United States of America; Georgetown University Medical Center, UNITED STATES

## Abstract

**Background:**

Rates of depression and addiction have risen drastically over the past decade, but the lack of integrative techniques remains a barrier to accurate diagnoses of these mental illnesses. Changes in reward/aversion behavior and corresponding brain structures have been identified in those with major depressive disorder (MDD) and cocaine-dependence polysubstance abuse disorder (CD). Assessment of statistical interactions between computational behavior and brain structure may quantitatively segregate MDD and CD.

**Methods:**

Here, 111 participants [40 controls (CTRL), 25 MDD, 46 CD] underwent structural brain MRI and completed an operant keypress task to produce computational judgment metrics. Three analyses were performed: (1) linear regression to evaluate groupwise (CTRL v. MDD v. CD) differences in structure-behavior associations, (2) qualitative and quantitative heatmap assessment of structure-behavior association patterns, and (3) the k-nearest neighbor machine learning approach using brain structure and keypress variable inputs to discriminate groups.

**Results:**

This study yielded three primary findings. First, CTRL, MDD, and CD participants had distinct structure-behavior linear relationships, with only 7.8% of associations overlapping between any two groups. Second, the three groups had statistically distinct slopes and qualitatively distinct association patterns. Third, a machine learning approach could discriminate between CTRL and CD, but not MDD participants.

**Conclusions:**

These findings demonstrate that variable interactions between computational behavior and brain structure, and the patterns of these interactions, segregate MDD and CD. This work raises the hypothesis that analysis of interactions between operant tasks and structural neuroimaging might aide in the objective classification of MDD, CD and other mental health conditions.

## Introduction

Mental health disorders, such as substance use disorder (SUD) and major depressive disorder (MDD), can cause significant morbidity and decreased quality of life [1–5]. While significant efforts have been made to better understand their underlying pathophysiology, they remain major health issues with complex symptom constellations [6, 7].

Clinically, MDD is diagnosed using qualitative questionnaires and symptom assessment. However, the constellation of symptoms meeting diagnostic criteria can vary greatly between patients. Research has also shown that the brain regions implicated in MDD are inconsistent across patients which may 1) reflect the diversity of symptom constellations and 2) make it difficult to study and diagnose with neuroimaging approaches [8]. Given this heterogeneity, some suggest that MDD represents a syndrome with significant variability, making accurate diagnosis and pathophysiology studies more difficult [6, 9, 10].

SUD is another complex condition that can share many symptom profiles with MDD [1, 11, 12]. One type of SUD involves cocaine dependence with polysubstance abuse (e.g., use with opioids; henceforth referred to as CD). Because of its propensity for co-morbidities, CD can also present with diverse symptoms and complex neurophysiology, ultimately hindering effective diagnosis and understanding of its pathophysiology [7, 13]. Together, the heterogeneity of MDD and propensity for CD co-morbidities, like MDD, contribute to their complexity and raise the question of how they may be quantitatively distinct.

Some hypothesize that cognitive science constructs that focus on approach/avoidance behavior or reward/aversion judgment may aide the characterization of complex mental health conditions like MDD and CD [14–16]. Such views have been indirectly supported by efforts such as the Research Domain Criteria (RDoC) project that focused two of its five main behavioral constructs around positive (approach) and negative (avoid) valence processes [17]. In behavioral neuroscience, operant keypressing is a gold-standard framework for quantifying approach and avoidance [18] in humans [19–22], which underpin reward and aversion judgments. Keypress behavior produces discrete and replicable patterns from which interpretable reward/aversion features, such as loss aversion, can be extracted [23–26]. Reward/aversion abnormalities [19, 27–30], and related structural and functional differences in the brain regions involved in reward/aversion judgment [19, 29, 31–34], have been implicated in the neuropathology of MDD and CD.

To our knowledge, no studies have investigated statistical interactions between operant behavior and related brain regions in humans to pinpoint quantitative differences across psychiatric illnesses (e.g., [8]). Here, we studied interactions between operant keypress metrics and brain morphometry to evaluate the effectiveness of three approaches to discriminate between healthy control (CTRL), MDD, and CD participants: (1) linear regression analysis to assess the variability of interactions between brain structures and keypress behavior metrics, henceforth referred to as structure-behavior interactions, across the three groups, (2) qualitative and quantitative heatmap analyses to assess structure-behavior slope differences across the three groups, and (3) machine learning (ML) with data fusion and a k-Nearest Neighbor approach (kNN) to assess group classification accuracy. Quantitatively defining distinctions between groups using variable interactions might yield useful signatures to objectively characterize these mental health conditions. These methods might then be applied to other complex disorders, with the ultimate goal of quantitively defining psychiatric conditions using simple cognitive science tasks and automated neuroimaging.

## Materials and methods

### Participant recruitment

Participants were recruited for the Massachusetts General Hospital (MGH) Phenotype Genotype Project (PGP) in Addiction and Mood disorders [35]. The PGP was approved by the MGH (Partners Human Research Committee) Institutional Review Board in accordance with the Declaration of Helsinki and all participants provided written, informed consent prior to participation. Details regarding recruitment of 77 CTRL, 47 MDD, and 120 CD have been previously published [36, 37] and are detailed in the Supplemental Material. For this study, data were excluded if (1) both the approach and avoidance keypress graphs for **KH**, **K*σ***, or **H**^**+**^**H**^**-**^ functions could not be produced or statistically fit (see S1 Appendix) or (2) structural brain data contained residual motion artifacts following motion correction (see S1 Appendix). Data exclusion resulted in 111 participants with complete data [40 CTRL, 46 CD, and 25 MDD (age = 37.88 ± 10.63 years)]. Participants were predominately white, right-handed males (S1A and S1B Table), with some demographic differences by group for gender, age, ethnicity, and years of education (S1 Appendix, S2 Table).

### Keypress task

An approach/avoidance keypress task was implemented using MATLAB as thoroughly described in [23]. This task quantifies the amount of work, in units of keypresses, that participants trade for picture viewing time. See S1 Appendix for further details.

### Magnetic resonance imaging

High-resolution T_1_-weighted structural, whole-brain magnetic resonance imaging (MRI) was conducted using a Siemens Avanto 1.5 T scanner (Siemens; Malvern, Pennsylvania) at the MGH Martinos Center (Charleston, MA). Acquisition details can be found in the S1 Appendix.

### Morphometric and topological brain structure analysis

Segmentation and normalization of 29 brain volumes (including right (R) and left (L) volumes, separately) is detailed elsewhere [36, 38–49] and summarized in the S1 Appendix. This study quantified large cortical volumes and standard subcortical structures using an anatomist-supervised, double-blind segmentation framework [36]. All brain volumes were normalized to total brain volume per participant. Brief descriptions of the brain volumes can be found in S3 Table.

### Statistical analyses

All statistical analyses were performed in STATA [50] apart from heatmap analyses which were performed in R [51].

#### Descriptive statistical measures from keypress task

Keypress data were modeled using relative preference theory (RPT), a framework that produces mathematically discrete, recurrent, robust, non-trivial, and scalable patterns [23, 26]. Details of the RPT-based variables and graph features follow prior procedures [23, 35, 36, 52] and are described in the S1 Appendix. This approach allows more than a dozen features to be quantified to facilitate psychological interpretation of human approach and avoidance judgment [52]. For this study, nine RPT features were used based on data completeness (i.e., there was insufficient data for plotting positive components of the RPT graphs).

**Fig 1:**
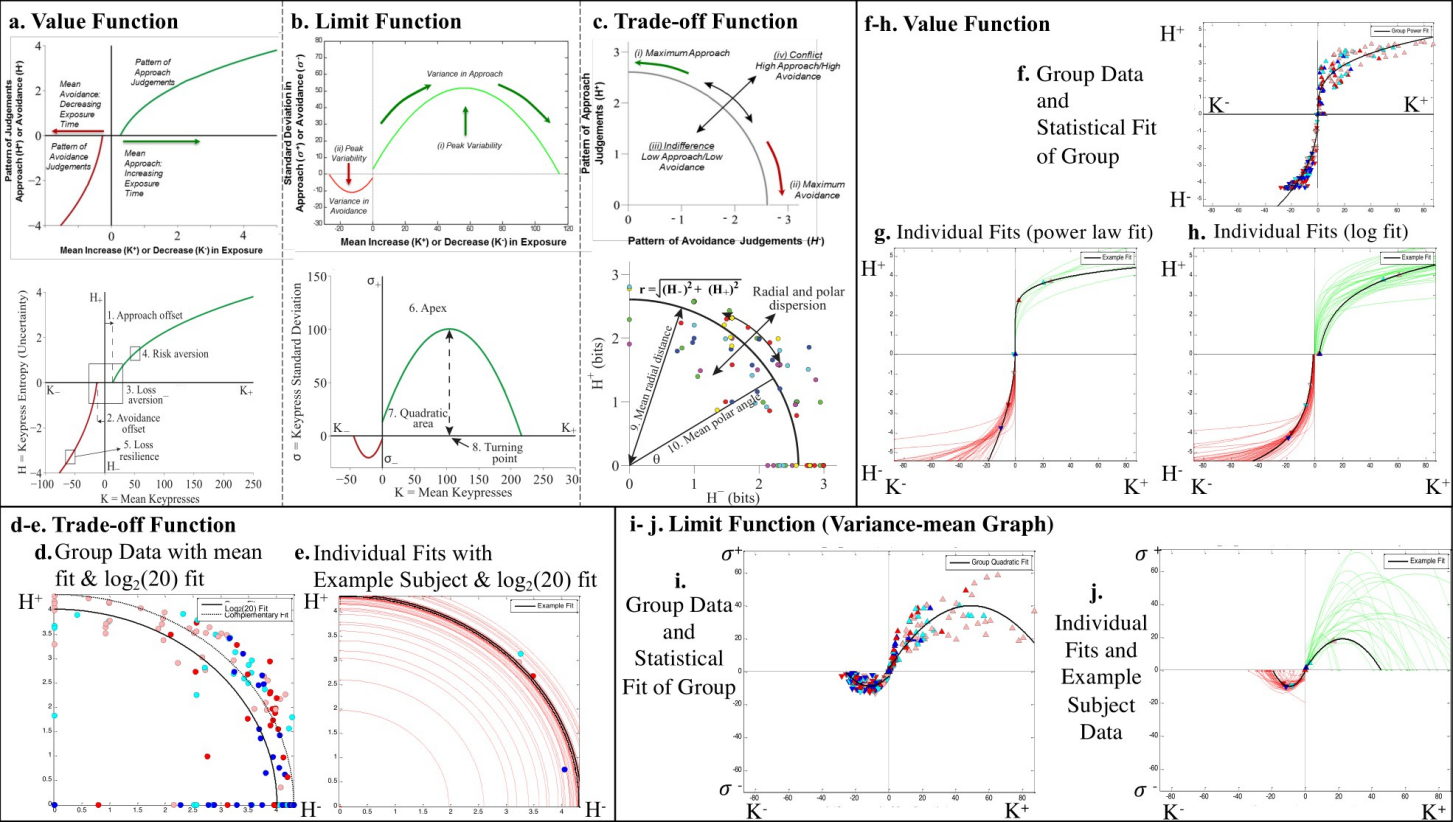
Relative preference theory. RPT is characterized in part by features that describe relationships between *a set of* behavioral variables: {*K*, *H*, *σ*}. These relationships include: **(a)** a value function plotting the Shannon entropy (*H*_±_), against the average ratings (*K*_±_) for approach or avoidance toward a set of objects. This function is referred to as a value function given it calibrates “wanting” or “liking” (depending on the task structure) against the pattern of previous judgments and resembles the prospect theory value function. Standard features of these curves, shown in the *bottom of the two* diagrams in (a), include loss aversion and risk aversion from the literature on behavioral economics. The corollary of risk aversion is also shown, herein referred to as loss resilience. Two offsets are also noted that are clear in the individual data, relating to an “approach offset” and “avoidance offset”. **(b)** A variance-mean relationship is observed between the average ratings (*K*_±_) plotted against the corresponding standard deviation of rating responses (*σ*_±_). This relationship is characterized by increasing variance up to a peak followed by decreasing variance back to baseline. This function describes *“*limits*”* or its “saturation”. Standard features of this curve include the apices of the quadratic fits, the “turning points” or value of *K*_±_ at which *σ*_±_ is maximal/minimal, and the quadratic areas (*QA*_±_) of these curves bounded by the *K*-axis. These curves resemble the variance-mean graphs of portfolio theory. **(c)** A trade-off function between the approach information (*H*_+_) and avoidance information (*H*_−_) can also be identified, defining how bundles of approach judgments were balanced with bundles of avoidance judgments as a quantifiable trade-off between *the patterns of* approach and avoidance judgment. This trade-off function can be characterized by the mean polar angle of the trade-off curves (*θ*), the standard deviation of this polar angle (its dispersion) (*σ*_*θ*_), the mean radial distance for the trade-off curves (*r*), and its corresponding standard deviation (the dispersion in r) (*σ*_*r*_). **(d)** Trade-off plot comparing approach information (*H*_+_) and avoidance information (*H*_−_) for keypressing across four picture categories in all subjects. The dotted black line denotes r = log_2_(20) for the number of pictures in each category used for the keypress task. The radial fit for group data was: $r=\sqrt{{\left(H_{+}\right)}^{2}+{\left(H_{-}\right)}^{2}}$. Note that pink dots are for model women faces, red dots are for non-model women faces, light blue dots are for model male faces, and dark blue dots are for non-model male faces. **(e)** Individual trade-off data for the four categories of picture are graphed as red lines for $r=\sqrt{{\left(H_{+}\right)}^{2}+{\left(H_{-}\right)}^{2}}$. One exemplar subject has their four data points shown, using the same color schema used for the group data as described in (d). Note that the theoretic log_2_(20) fit sits just inside the fit for the exemplar subject. **(f)** Value function comparing mean keypress intensity (*K*) to keypress information (*H*) across the four picture categories in all subjects. The group fit using a power law function is shown as a black line against individual data as triangles using the same color schema as in (d). **(g) and (h)** Individual value function graphs are shown with power law fits (g) and log fits (h). Note the ease of fitting with x-axis offsets for the log functions in (h). In each graph, one exemplar subject has their four data points shown, using the same color schema used for the group data as described in (d). Approach graphs (*K*_+_,*H*_+_) are shown in green, whereas avoidance (*K*_−_,*H*_−_) graphs are shown in red. **(i)** Limit function comparing *K* to the standard deviation of approach or avoidance keypressing (*σ*) across picture categories in group data. Note the group fit is a quadratic function for both approach and avoidance (black parabolic line). **(j)** Approach and avoidance data for individual participants were fit to quadratic functions, where green fits represented positively valenced keypresses, and red fits represented negatively valenced keypresses.

The nine features extracted from RPT graphs are illustrated in Fig 1 and are computationally described in S1 Appendix. Features included loss resilience, negative offset, negative apex, negative turning point, negative quadratic area (henceforth, negative area), mean polar angle (polar angle), polar angle standard deviation (polar dispersion), mean radial distance (radial distance), and radial distance standard deviation (radial dispersion), Additionally, the six variables used to mathematically graph RPT features were also included, as described in detail elsewhere [19, 20, 23–26, 35, 36]: mean number of keypresses to increase viewing time (henceforth, **K+**), mean number of keypresses to decrease viewing time (**K-**), mean Shannon entropy or uncertainty for positive keypresses (**H+**), mean Shannon entropy or uncertainty for negative keypresses (**H-**), standard deviation for positive keypresses (***σ*+**), and standard deviation for negative keypresses (***σ*-**). The nine RPT features, and six variables used to derive these features, are henceforth referred to as “keypress metrics”.

#### Linear regression

The 15 keypress metrics were regressed against 29 brain volumes, using Cook’s distance for outlier removal [53, 54], to investigate the variability of associations between structure and behavior across CTRL, MDD, and CD. Prior to regression analysis, brain volumes were analyzed against demographic variables, by group, to determine which covariate(s) to include in each model. Although brain volumes were normalized to each participant’s total brain volume, which should correct for demographic variance, it is possible differences could still exist. Brain volumes were thus analyzed (1) by ethnicity using the Wilcoxon Rank Sum test (*α* = .05) [55] (note: participants identifying as Asian were dropped from analyses given the small sample across groups) and (2) by age and years of education using the Spearman correlation test (*α* = .05) [56]. If *p* < .05, the demographic variable was included in the regression model.

Regressions were run for each group (CTRL, MDD, CD) with keypress metrics as the dependent variable and brain volumes as the independent variable, with or without covariates. Regressions were considered significant for *p*-value < .05. The Benjamini-Hochberg multiple comparison correction [57] was applied over the 15 keypress metrics to compute *q*-values. Standardized beta coefficients (Std. *β*_*volume*_), 95% confidence intervals (95% CI_volume_), *p*-values, *q*-values (*q*_*Hochberg*_), and the number of Cook’s distance outliers removed (# outliers) were reported. From these regressions, brain regions that were common across groups were noted. Regression relationships that were common between groups were also noted.

#### Heatmap assessment

Beta (*β*) coefficients (i.e., slopes) were further investigated for group differences using a covariance matrix, or heatmap, approach. Here, *β* coefficients were calculated *without* the inclusion of covariates given (1) steps were taken to minimize demographic influence (i.e., brain volume normalization), and (2) the goal of streamlining the analysis (given covariates were only required for a minority, or subset, of the regression models described above). Heatmaps were generated to visualize the magnitude of the *β* coefficients for each participant in each group (range = -1 to +1). Four types of heatmaps were generated: (1) *β* coefficients from structure-behavior regressions, (2) difference in absolute magnitude of *β* coefficients between groups (e.g., |*β*_*CTRL*_|−|*β*_*MDD*_|; this quantifies valence), (3) absolute value of the difference between *β* coefficients (e.g., |*β*_*CTRL*_−*β*_*MDD*_|; this quantifies intensity), and (4) *β* directionality agreeance (i.e., both negative/positive versus one positive and one negative) between *β* coefficients where -1 equates to opposite and +1 equates to the same *β* directionality between two groups. Heatmaps were plotted using the ‘heatmap.2’ command from the ‘gplots’ open access R package.

Three statistical tests were used to determine if there were overall differences in *β* values between groups. First (from heatmap (2), above), *t*-tests were used to assess if the mean difference between the overall absolute magnitude of the *β* coefficients was zero between groups (distributions were Gaussian; *α* = .05; value range = -1-1). The null hypothesis was that the mean of the error terms equaled zero. Second (from heatmap (3), above), the Wilcoxon Rank Sum test (*α* = .05; value range = 0–2) was used to assess the absolute difference between *β* coefficients (distributions were not Gaussian). Wilcoxon Rank Sum test tested for overall differences where the null hypothesis was that the median of the error distribution was zero. Third (from heatmap (4), above), a proportion test [58] was implemented to investigate whether the directions of the *β* coefficients were the same (i.e., both positive or both negative) between two groups. If the *β* coefficients had the same directionality, this was deemed a success, and the null hypothesis was defined as a probability of a success being .5.

#### Selection of metrics for k-nearest neighbor analyses

Keypress metrics, brain volumes, and demographic variables were assessed for differences across the three groups (CTRL, MDD, CD). Keypress metric differences were assessed using the Dunn test (*α* = .05) [59]. Significant results (*p* < .05) were corrected for multiple comparisons using the Benjamini-Hochberg procedure to obtain *q*-values; *q*-values < .05 were considered significant. Brain volumes were analyzed with the Dunn test and significant results (*p* < .05) were corrected for multiple comparisons using the Benjamini-Hochberg procedure to obtain *q*-values (*α* = .05). Demographic variables from S2 Table were included.

#### k-Nearest neighbor (kNN)

Significant results from the groupwise comparisons, above, were selected for the k-Nearest Neighbor (kNN) leave-one-out classification approach [60–62] to classify CTRL from MDD, CTRL from CD, and MDD from CD. The kNN analysis was run using six variable combinations: (1) keypress metrics only, (2) keypress metrics and demographic variables, (3) brain volumes only, (4) brain volumes and keypress metrics, (5) brain volumes and demographic variables, and (6) keypress metrics, brain volumes, and demographic variables. The number of nearest neighbors (***k***) was set to 5, a Mahalanobis transform was applied to transform continuous data before computing the dissimilarities, and prior proportions were applied (e.g., 61.5% CTRL and 38.5% MDD). Further details are described in S1 Appendix.

#### *Post-hoc* assessment: Impulsivity and keypress behavior

The Temperament and Character Inventory (TCI) test, which included a novelty seeking score, was completed by 53 of the 111 participants. Novelty seeking is higher in individuals who are more impulsive. To investigate the potential relationship between impulsivity and keypressing behaviors, linear relationships between the 15 keypress metrics and novelty seeking were assessed (*α* = .05) using Cook’s distance for outlier removal. This was done for the combined cohort (n = 53) as well as for each cohort independently [CTRL (n = 19), MDD (n = 19), and CD (n = 15)].

## Results

We used three approaches to assess how well variable interactions [(1) structure-behavior regressions, (2) heatmap analysis], or variable fusion [(3) kNN prediction], facilitated segregation of clinical groups. Results from these three approaches are described independently.

### Covariate assessment and structure-behavior regressions

Three demographic variables showed effects for brain volume (ethnicity, age, and years of education) and were included as covariates. Eleven brain volumes differed by ethnicity across CTRL, MDD, and CD groups (Wilcoxon Rank Sum test *p* < .05; S4A Table). Twenty-one brain volumes differed by age across groups (Spearman *p* < .05; S4B Table). Two brain volumes differed by years of education in CTRL (Spearman *p* < .05; S4C Table).

**Fig 2:**
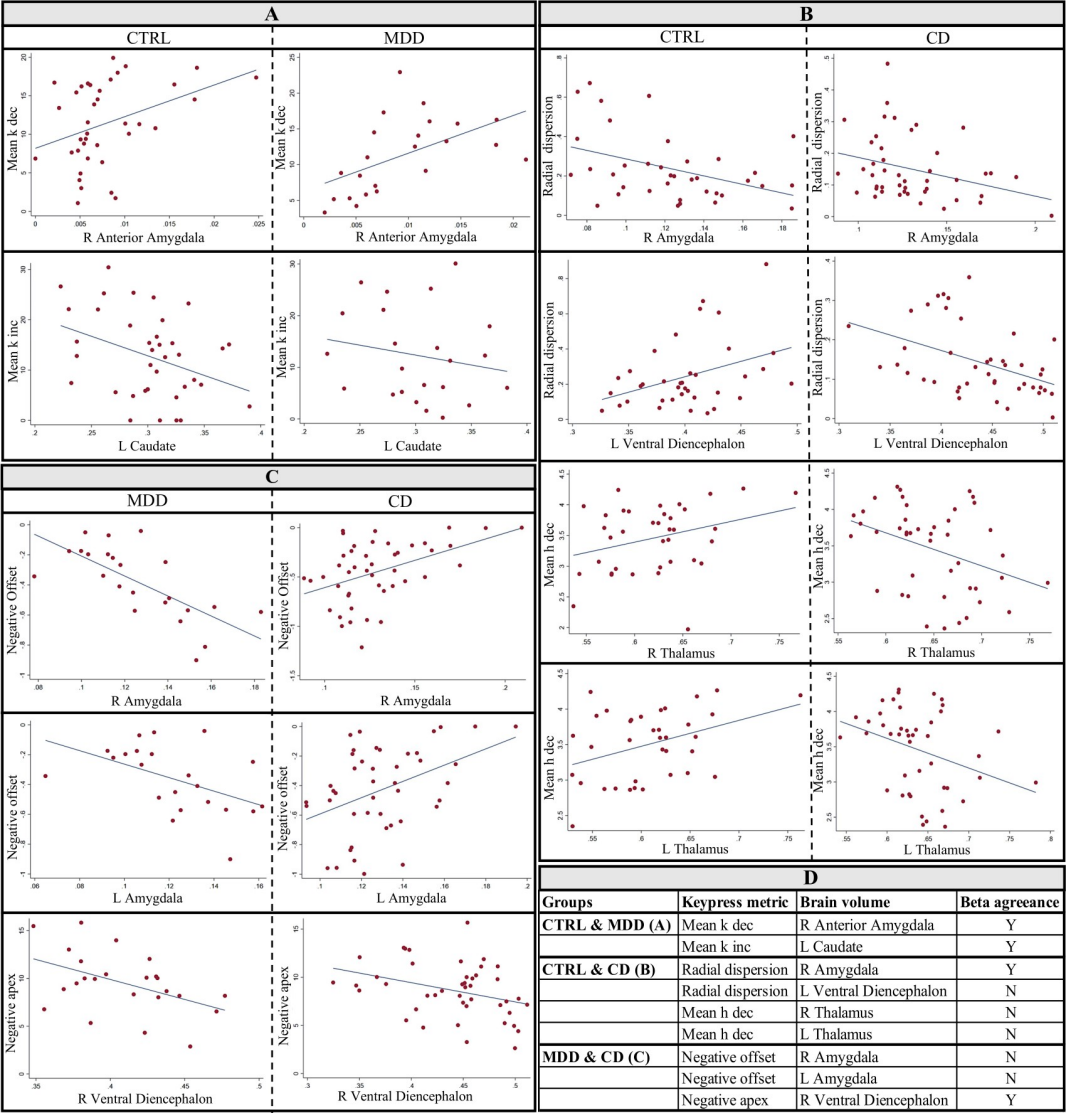
Assessment of overlapping regressions between CTRL, MDD, and CD. Keypress metrics are plotted on the y-axis and brain volumes are plotted on the x-axis. Regressions are plotted based on results presented in Table 1 with inclusion of covariates. (A) Two regressions were common between CTRL and MDD with slope in the same direction. (B) Four regressions were common between CTRL and CD but only one common pair had slope terms in the same direction. (C) Three regressions were common between MDD and CD but only one common pair had slope terms in the same direction. (D) A summary table of common regressions between CTRL and MDD, CTRL and CD, and MDD and CD. Beta agreeance indicates if the slope terms were in the same direction.

With the inclusion of covariates as per S4 Table, there were 39 structure-behavior relationships in CTRL, 28 in MDD, and 35 in CD (*p* < .05, Table 1, S1 Fig). Of these 102 regressions, only two pairs of regressions were common between CTRL and MDD, four pairs between CTRL and CD, and three pairs between MDD and CD (Fig 2). Of these nine pairs of common regressions, five pairs had *β* terms in opposing directions (i.e., positive vs. negative); thus, of the 102 significant regressions between keypress metrics and brain volumes across the three groups, only four pairs of regressions were consistent (8 of 102 = 7.8%), with a *β* term in the same direction (both positive *or* negative). Within group, application of multiple comparisons correction indicated seven significant regressions (*q*_*Hochberg*_ < .05) for CTRLs, three for MDD, and four for CD. Of these 14 regressions, only one pair (2 of 14 = 14%) was consistent between MDD and CD (negative offset vs. right amygdala).

**Table 1:** Structure-behavior regression analysis results with covariate inclusion for (A) CTRL, (B) MDD, and (C) CD groups.

**(A) CTRL**^**a**^ **(n = 40; n = 39 when education included in model)**																		
**Keypress metric (Y)**		**Brain Volume (X)**		**Covariate(s)**			***p***		***q***_***Hochberg***_			**Std. *β***			**95% CI**			**# outliers**
Loss Resilience		R Cerebral White Matter		Age			.036		.504			-0.3776			-0.7297, -0.0256			2
Negative Offset		L Cerebral White Matter		Age			.041		.574			-0.3728			-0.7286, -0.0170			2
Negative Apex		R Caudate		Age			.040		.480			-0.3754			-0.7336, -0.0172			2
Negative Turning Point		R Caudate		Age			.011		.165			-0.4496			-0.7899, -0.1090			2
Negative Turning Point		L Caudate		Age			.026		.390			-0.4087			-0.7661, -0.0513			2
Negative Turning Point		R Hippocampus		--			.014		.210			0.3942			0.0835, 0.7048			2
Negative Turning Point		L Hippocampus		--			.041		.615			0.3338			0.0152, 0.6525			2
Radial Dispersion		R Caudate		Age			.037		.480			0.3863			0.0243, 0.7483			3
Radial Dispersion		R Amygdala		--			.012		.180			-0.4100			-0.7229, -0.0970			3
Radial Dispersion		L Ventral Diencephalon		--			.021		.315			0.3775			0.0597, 0.6952			3
Polar Angle		L Cerebellum White Matter		Ethnicity			.039		.546			-0.3647			-0.7096, -0.0197			4
Polar Angle		R Thalamus		Education			.011		.154			-0.5217			-0.9133, -0.1300			2
**Polar Angle**		**L Thalamus**		**Education**			**.001**		**.014**			**-0.5912**			**-0.9309, -0.2515**			**3**
Polar Angle		R Putamen		Age			.015		.195			-0.4134			-0.7406, -0.0862			2
Polar Angle		L Putamen		Age			.005		.070			-0.4794			-0.8002, -0.1586			3
**Polar Dispersion**		**R Thalamus**		**Education**			**.002**		**.030**			**-0.5931**			**-0.9474, -0.2388**			**4**
**Polar Dispersion**		**L Thalamus**		**Education**			**.001**		**.014**			**-0.6119**			**-0.9612, -0.2625**			**5**
Polar Dispersion		R Ventral Diencephalon		--			.041		.615			-0.3335			-0.6521, -0.0148			2
Mean h dec		R Thalamus		Education			.020		.240			0.4388			0.0728, 0.8047			2
**Mean h dec**		**L Thalamus**		**Education**			**.003**		**.036**			**0.5586**			**0.2020, 0.9153**			**3**
Mean h inc		L Cerebellum White Matter		Ethnicity			.048		.624			-0.3500			-0.6974, -0.0025			4
Mean h inc		L Thalamus		Education			.013		.143			-0.4751			-0.8439, -0.1064			2
Mean h inc		L Putamen		Age			.035		.420			-0.3705			-0.7143, -0.0267			3
Mean k dec		L Cerebellum Cortex		--			.048		.720			-0.3267			-0.6510, -0.0024			3
Mean k dec		R Amygdala (Anterior)		--			.022		.330			0.3697			0.0557, 0.6838			2
Mean k inc		R Cerebellum White Matter		Age, Ethnicity			.033		.462			-0.4170			-0.7975, -0.0365			5
Mean k inc		L Cerebellum White Matter		Ethnicity			.020		.300			-0.4213			-0.7705, -0.0721			5
Mean k inc		R Thalamus		Education			.019		.240			-0.4575			-0.8337, -0.0813			1
**Mean k inc**		**L Thalamus**		**Education**			**.003**		**.036**			**-0.5251**			**-0.8635, -0.1867**			**2**
Mean k inc		R Caudate		Age			.039		.480			-0.4132			-0.8052, -0.0212			4
Mean k inc		L Caudate		Age			.032		.448			-0.3981			-0.7602, -0.0359			2
**Mean k inc**		**R Putamen**		**Age**			**.003**		**.045**			**-0.4841**			**-0.7947, -0.1736**			**1**
**Mean k inc**		**L Putamen**		**Age**			**.001**		**.015**			**-0.5283**			**-0.8370, -0.2196**			**1**
Mean k inc		Brain stem		Ethnicity			.027		.405			-0.4321			-0.8111, -0.0531			5
Mean std dec		R Cerebral White Matter		Age			.014		.210			0.43658			0.0956, 0.7776			2
Mean std dec		L Cerebral White Matter		Age			.006		.090			0.4712			0.1458, 0.7965			2
Mean std dec		R Cerebellum White Matter		Age, Ethnicity			.028		.420			0.4003			0.0466, 0.7539			2
Mean std inc		R Putamen		Age			.012		.168			-0.4273			-0.7545, -0.10000			2
Mean std inc		L Putamen		Age			.016		.208			-0.4188			-0.7536, -0.0839			2
**(B) MDD**^**b**^ **(n = 25)**																		
**Keypress metric (Y)**	**Brain Volume (X)**		**Covariate(s)**			***p***					***q***_***Hochberg***_			**Std. *β***			**95% C.I.**	**# outliers**
Loss Resilience	R Pallidum		--			.018					.252			-0.4785			-0.8667, -0. 0902	1
Loss Resilience	L Pallidum		--			.004					.056			-0.5745			-0.9459, -0.2030	2
Negative Offset	R Cerebellum (Exterior)		--			.022					.330			-0.4758			-0.8750, -0.0767	2
Negative Offset	R Cerebellum Cortex		--			.016					.240			-0.4972			-0.8909, -0.1034	2
Negative Offset	R Thalamus		--			.007					.105			-0.5329			-0.9071, -0.1588	1
**Negative Offset**	**L Thalamus**		**--**			**.001**					**.015**			**-0.6410**			**-0.9893, -0.2926**	**2**
Negative Offset	R Pallidum		--			.008					.120			-0.5288			-0.9041, -0.1535	1
**Negative Offset**	**L Pallidum**		**--**			**.003**					**.045**			**-0.5873**			**-0.9546, -0.2200**	**2**
**Negative Offset**	**R Amygdala**		**--**			**< .001**					**.003**			**-0.6997**			**-1.0239, -0.3754**	**2**
Negative Offset	L Amygdala		--			.021					.315			-0.4884			-0.8954, -0.0814	3
Negative Apex	R Thalamus		--			.033					.462			0.4554			0.0402, 0.8707	3
Negative Apex	L Thalamus		--			.021					.294			0.4888			0.0818, 0.8957	3
Negative Apex	R Ventral Diencephalon		--			.022					.308			-0.4639			-0.8556, -0.0722	1
Radial Distance	L Cerebellum White Matter		--			.044					.660			-0.4244			-0.8353, -0.0135	2
Radial Dispersion	L Amygdala		--			.046					.644			0.4397			0.0085, 0.8710	4
Polar Angle	Brain stem		--			.029					.435			-0.4558			-0.8597, -0.0519	2
Mean h dec	R Nucleus Accumbens		--			.013					.195			0.5001			0.1172, 0.8830	1
Mean h dec	R Ventral Diencephalon		--			.008					.120			-0.5387			-0.9210, -0.1563	2
Mean h dec	L Ventral Diencephalon		--			.013					.195			-0.5087			-0.8994, -0.1180	2
Mean h inc	R Cerebellum White Matter		--			.027					.405			-0.4604			-0.8633, -0.0576	2
Mean k dec	Brain stem		--			.046					.644			0.4105			0.0073, 0.8137	1
Mean k dec	R Amygdala (Anterior)		--			.009					.135			0.5295			0.1445, 0.9145	2
Mean k dec	R Nucleus Accumbens		--			.016					.224			0.4878			0.1019, 0.8738	1
Mean k inc	L Caudate		Age			.043					.645			-0.4800			-0.9424, -0.0177	2
Mean k inc	L Nucleus Accumbens		Age			.039					.546			-0.4559			-0.8875, -0.0242	2
Mean std inc	R Cerebellum Cortex		--			.046					.644			0.4118			0.0089, 0.8147	1
Mean std inc	L Cerebellum Cortex		--			.042					.630			0.4281			0.0179, 0.8382	2
Mean std inc	L Nucleus Accumbens		Age			.036					.540			-0.4595			-0.8867, -0.0324	2
**(C) CD**^**c**^ **(n = 46)**																		
**Keypress metric (Y)**		**Brain Volume (X)**			**Covariate(s)**			***p***		***q***_***Hochberg***_			**Std. *β***			**95% C.I.**		**# outliers**
Loss Resilience		R Thalamus			--			.005		.075			-0.4152			-0.6985, -0.1319		2
Loss Resilience		L Thalamus			--			.015		.225			-0.3644			-0.6544, -0.0745		2
Loss Resilience		R Ventral Diencephalon			--			.021		.273			0.3509			0.0556, 0.6463		3
Negative Offset		L Hippocampus			--			.031		.465			0.3296			0.0319, 0.6274		3
**Negative Offset**		**R Amygdala**			**--**			**.002**		**.030**			**0.4528**			**0.1786, 0.7270**		**1**
Negative Offset		L Amygdala			--			.005		.070			0.4189			0.1361, 0.7016		2
Negative Apex		L Pallidum			--			.026		.390			0.3391			0.0424, 0.6358		3
Negative Apex		R Amygdala			--			.007		.098			-0.4043			-0.6928, -0.1158		3
Negative Apex		L Amygdala			--			.005		.070			-0.4211			-0.7071, -0.1350		3
Negative Apex		R Ventral Diencephalon			--			.023		.276			-0.3420			-0.6346, -0.0493		2
Radial Distance		R Cerebral White Matter			Age			.050		.750			0.3062			-0.0002, 0.6126		2
Radial Distance		R Cerebral Cortex			Age			.016		.240			-0.3621			-0.6522, -0.0180		2
Radial Distance		L Cerebral Cortex			Age			.037		.518			-0.3325			-0.6430, -0.0219		3
Radial Dispersion		R Pallidum			Age			.016		.240			0.4220			0.0814, 0.7625		3
Radial Dispersion		R Amygdala			--			.036		.396			-0.3130			-0.6051, -0.0209		1
Radial Dispersion		R Ventral Diencephalon			--			.006		.084			-0.4141			-0.7011, -0.1270		3
**Radial Dispersion**		**L Ventral Diencephalon**			**--**			**.003**		**.045**			**-0.4471**			**-0.7293, -0.1650**		**3**
**Polar Dispersion**		**L Caudate**			**--**			**.003**		**.045**			**-0.4441**			**-0.7267, -0.1616**		**3**
**Polar Dispersion**		**R Nucleus Accumbens**			**--**			**< .001**		**.015**			**-0.4931**			**-0.7675, -0.2187**		**3**
Polar Dispersion		L Nucleus Accumbens			--			.008		.120			-0.4010			-0.6899, -0.1120		3
Mean h dec		R Cerebellum White Matter			--			.011		.165			0.3839			0.0926, 0.6751		3
Mean h dec		R Thalamus			--			.013		.169			-0.3702			-0.6595, -0.0809		2
Mean h dec		L Thalamus			--			.029		.377			-0.3290			-0.6231, -0.0349		2
Mean k dec		R Thalamus			--			.009		.126			-0.3905			-0.6772, -0.1039		2
Mean k dec		L Thalamus			--			.027		.377			-0.3296			-0.6200, -0.0393		1
Mean k inc		L Cerebral Cortex			Age			.024		.360			-0.3681			-0.6859, -0.0503		3
Mean k inc		R Amygdala			--			.011		.132			-0.3819			-0.6697, -0.0941		2
Mean k inc		L Amygdala			--			.026		.312			-0.3362			-0.6294, -0.0429		2
Mean std dec		R Caudate			--			.045		.675			0.3080			0.0079, 0.6080		3
Mean std dec		L Caudate			--			.036		.504			0.3214			0.0227, 0.6200		3
Mean std dec		R Ventral Diencephalon			--			.004		.060			-0.4282			-0.7096, -0.1467		2
Mean std dec		L Ventral Diencephalon			--			.009		.126			-0.3917			-0.6782, -0.1052		2
Mean std inc		R Amygdala			--			.011		.132			-0.3798			-0.6679, -0.0918		2
Mean std inc		L Amygdala			--			.011		.143			-0.3786			-0.6668, -0.0903		2
Mean std inc		R Amygdala (Anterior)			--			.017		.255			0.3626			0.0686, 0.6545		3

*p* = *p*-values (*α* = .05); boldface entries indicate regressions with *q*_*Hochberg*_ values less than .05; 95% C.I. = 95% confidence intervals; # outliers = the number of outliers removed based on Cook’s distance outlier procedure.

^a^CTRL = control participants

^b^MDD = major depressive disorder participants

^c^CD = cocaine dependence disorder participants

Excluding the nine overlapping regressions from the total 102, four brain regions overlapped between any two groups (excluding overlapping regressions presented in Fig 2 and Table 1): R Cerebral White Matter, R Ventral Diencephalon, R Cerebellum White Matter, and L Thalamus (S5 Table).

A small set of keypress metrics were unique to each group (Table 1). CTRL participants exhibited regressions with negative turning point, lacked regressions with radial distance, and the number of regressions with polar angle was more than double the number observed for MDD or CD. MDD lacked regressions for polar dispersion and ***σ*-** metrics, and the number of regressions with negative offset was more than double the number observed for CTRL or CD. CD alone lacked regressions for polar angle and **H**+ metrics. These differences alone distinguished the three groups.

### Heatmap analyses

**Fig 3:**
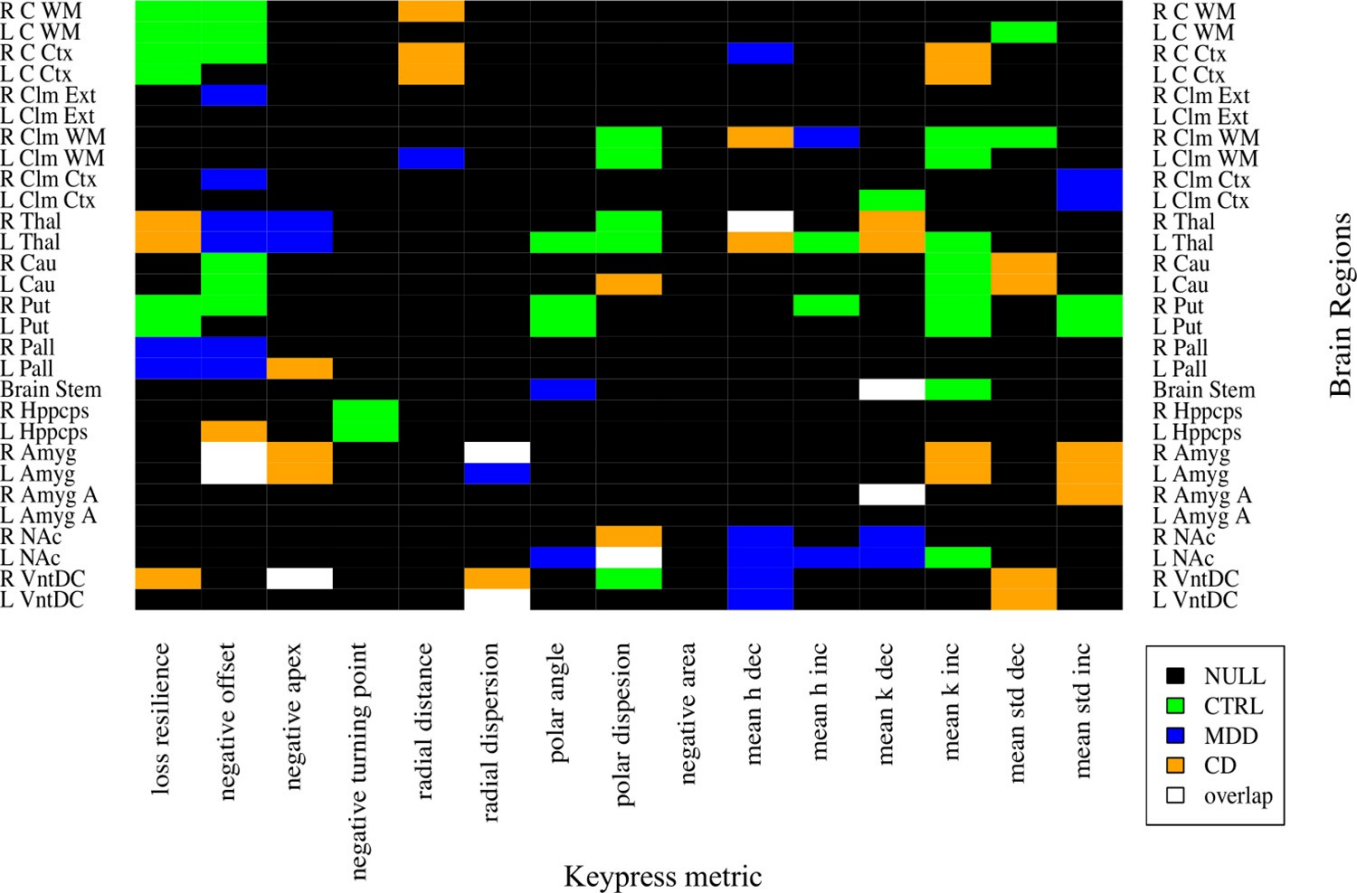
Heatmap presentation of structure-behavior regressions for CTRL, MDD, and CD. Brain volumes are labeled on the y-axis and keypress metrics on the x-axis. Values are plotted based on results in S6 Table and S2 Fig where covariates were not included in the regression models. Black cells indicate null regression results, green cells indicate significant regressions (*P* < .05) for CTRL, blue cells indicate significant regressions for MDD, and orange cells indicate significant regression for CD. The white cells indicate common regressions between any of the two groups.

For heatmap analyses, linear regressions between keypress metrics and brain volumes were completed without inclusion of covariates given (1) demographic influence was already minimized through brain volume normalization, and (2) covariate effects were only found in a minority of the regression models. *β* coefficients from these regressions (S6 Table) were used to build heatmaps for CTRL, MDD, CD and visualize regression overlap between groups (Fig 3). Of the 109 regressions, nine pairs overlapped between any two groups, and only four pairs exhibited slopes in the same direction (S2 Fig).

Excluding the nine overlapping regressions from the total 109, six brain regions overlapped between any two groups (excluding overlapping regressions presented in S2 Fig and S6 Table): R Cerebral Cortex, L Cerebral Cortex, R Cerebral White Matter, R Ventral Diencephalon, R Cerebellum White Matter, and L Thalamus (S7 Table).

**Fig 4:**
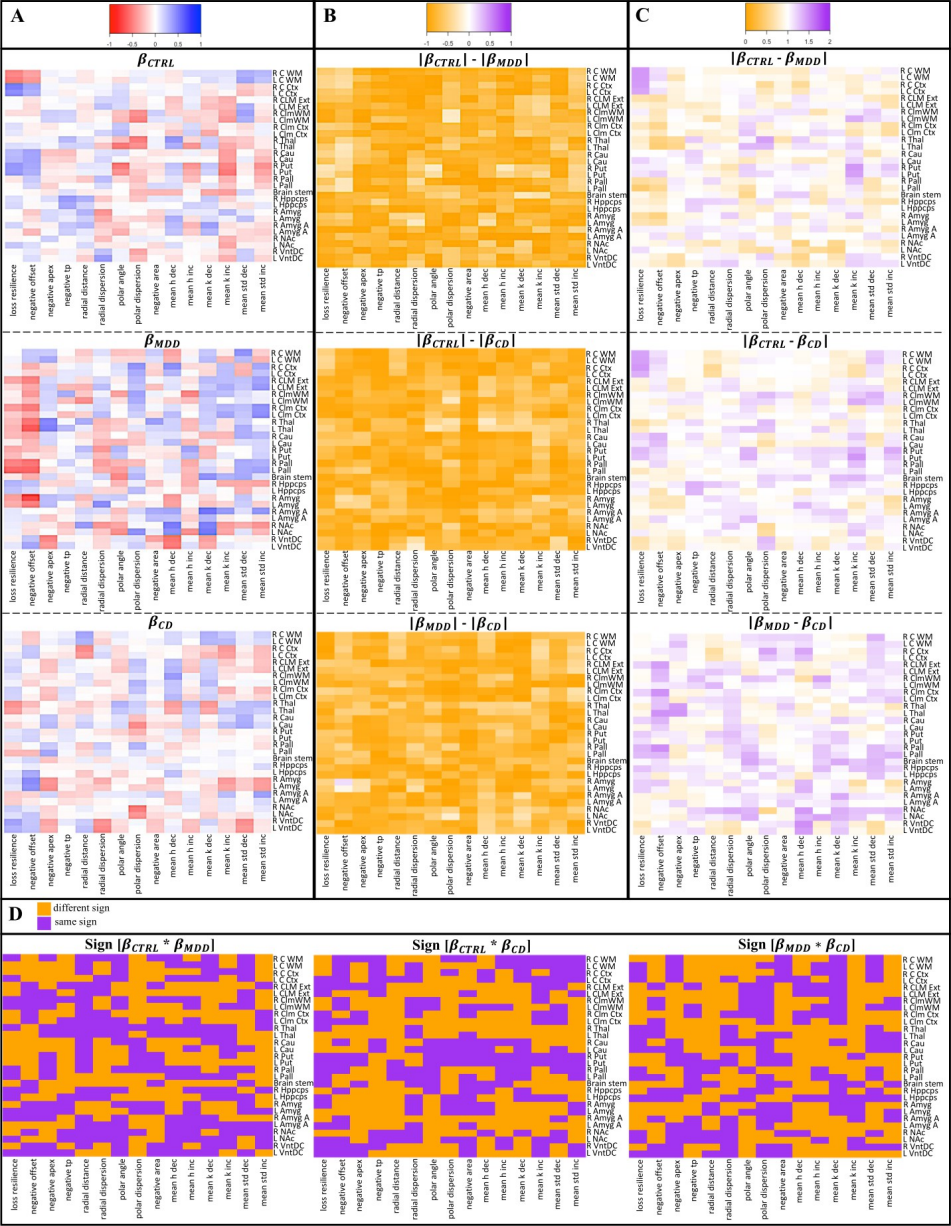
Heatmap assessment of slope (*β*) differences between CTRL, MDD, and CD. Brain volumes are labeled on the y-axis and keypress metrics on the x-axis. (A) 29 brain volumes and 15 keypress metrics were regressed without the inclusion of covariates to obtain *β* term values [range -1 (red) to +1 (blue)] for CTRL, MDD, and CD. (B) The difference between absolute *β* values are displayed on a scale of -1 (orange) to +1 (purple). (C) The absolute differences between *β* terms are given on a scale of 0 (orange) to 2 (purple). (D) Orange cells indicate that *β* term directionality differed between any two groups and purple indicates that *β* terms were in the same direction.

Heatmaps displaying *β* terms from structure-behavior regressions were qualitatively distinct for each group (Fig 4A). Heatmaps displaying the valence (Fig 4B) and intensity (Fig 4C) of *β* term differences between groups were also qualitatively distinct, as were differences in *β* term directionality (i.e., positive vs. negative slope; orange cells in Fig 4D). Three statistical tests were implemented to quantify *β* term differences between the three groups (see *Methods*: *Heatmap assessment*). *t*-Test assessment of *β* term valence revealed significant differences between CTRL and MDD (*p* < .001) as well as MDD and CD (*p* < .001) groups. Wilcoxon Rank Sum test assessment of *β* term intensity yielded significant differences between CTRL and MDD (*p* < .001; median = 0.179), CTRL and CD (*p* < .001; median = 0.203), and MDD and CD (*p* < .001; median = 0.216). Proportions tests of *β* term directionality differences showed there was a higher probability (>50%) of opposing *β* terms between MDD and CD (*p* = .0073). Results are summarized in Table 2.

**Table 2:** Statistical groupwise comparisons of *β* terms from structure-behavior regressions.

Statistical test	Group comparison	*p*-value	Median
***t*-test**	CTRL^a^ vs. MDD^b^	< .001	--
	CTRL vs. CD^c^	.166	--
	MDD vs. CD	< .001	--
**Wilcoxon Rank Sum**	CTRL vs. MDD	< .001	0.179
	CTRL vs. CD	< .001	0.203
	MDD vs. CD	< .001	0.216
**Proportions test**	CTRL vs. MDD	.924	--
	CTRL vs. CD	.055	--
	MDD vs. CD	.00725	--

Significance level (*α*) was .05 for all statistical tests.

^a^CTRL = control participants

^b^MDD = major depressive disorder participants

^c^CD = cocaine dependence disorder participants

### Feature selection from groupwise keypress and brain volume differences

One keypress metric, radial dispersion, differed across the three groups (Kruskal Wallis *p* = .0216). Overall, nine brain volumes differed across groups (*p* < .05; Table 3). Specifically, three volumes differed between CTRL and MDD, eight between CTRL and CD, and eight between MDD and CD (*q*-value < .05). Radial dispersion and the nine brain volumes were included in the kNN models.

**Table 3:** Assessment of brain volume differences between CTRL, MDD, and CD participants.

Volume	Comparison	*p*	*q*_*Hochberg*_
Right Cerebral White Matter	overall	< .001	--
	***CTRL***^***a***^ ***vs*. *MDD***^***b***^	**.*0421***	**.*0421***
	***CTRL vs*. *CD*** ^***c***^	***<* .*001***	***<* .*001***
	***MDD vs*. *CD***	**.*0117***	**.*0235***
Left Cerebral White Matter	overall	.0011	--
	CTRL vs. MDD	.1180	.1180
	***CTRL vs*. *CD***	***<* .*001***	***<* .*001***
	***MDD vs*. *CD***	**.*0248***	**.*0497***
Right Cerebral Cortex	overall	< .001	--
	***CTRL vs*. *MDD***	**.*0364***	**.*0364***
	***CTRL vs*. *CD***	***<* .*001***	***<* .*001***
	***MDD vs*. *CD***	**.*0118***	**.*0235***
Left Cerebral Cortex	overall	< .001	--
	CTRL vs. MDD	.0630	.0630
	***CTRL vs*. *CD***	***<* .*001***	***<* .*001***
	***MDD vs*. *CD***	**.*0090***	**.*0179***
Left Cerebellum (Exterior)	overall	.0498	--
	***CTRL vs*. *MDD***	**.*0182***	**.*0365***
	CTRL vs. CD	.4256	.4256
	***MDD vs*. *CD***	**.*0105***	**.*0314***
Right Cerebellar White Matter	overall	.0496	--
	CTRL vs. MDD	.3955	.3955
	***CTRL vs*. *CD***	**.*0201***	**.*0403***
	**MDD vs. CD**	**.0199**	.0596
Left Thalamus	overall	.0147	--
	CTRL vs. MDD	.4730	.4730
	***CTRL vs*. *CD***	**.*0044***	**.*0133***
	***MDD vs*. *CD***	**.*0136***	**.*0272***
Brain Stem	overall	.0249	--
	CTRL vs. MDD	.4956	.4956
	***CTRL vs*. *CD***	**.*0078***	**.*0235***
	***MDD vs*. *CD***	**.*0172***	**.*0345***
Right Ventral Diencephalon	overall	.0012	--
	CTRL vs. MDD	.3530	.3530
	***CTRL vs*. *CD***	***<* .*001***	***<* .*001***
	***MDD vs*. *CD***	**.*0048***	**.*0095***

Significant results (*p* < .05) were corrected for multiple comparisons to obtain *q*_*Hochberg*_.

^a^CTRL = control participants

^b^MDD = major depressive disorder participants

^c^CD = cocaine dependence disorder participants

### kNN classification

**Fig 5:**
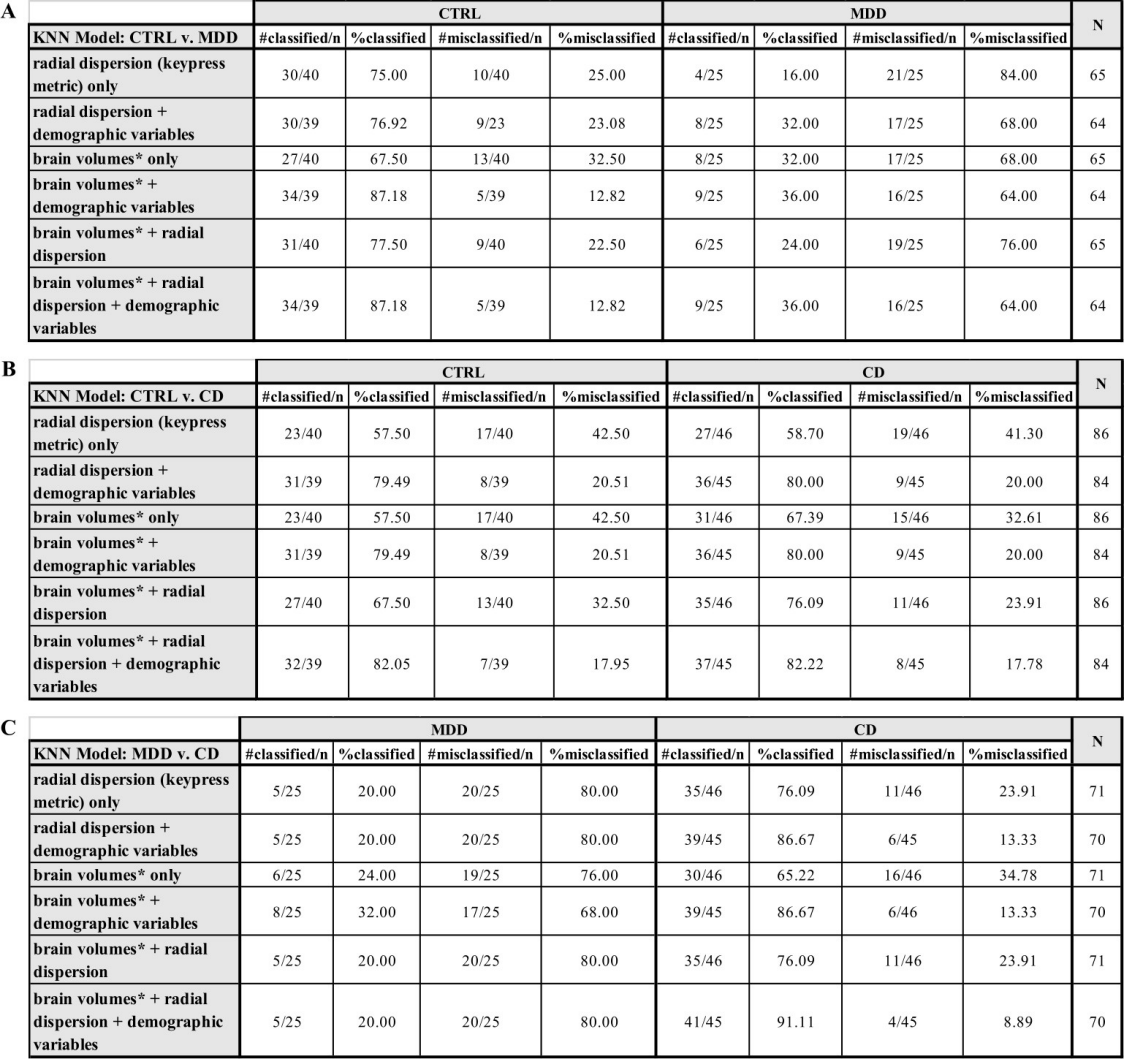
kNN results. (A) Classification results for CTRL and MDD. (B) Classification results for CTRL and CD. (C) Classification results for MDD and CD. RPT refers to relative preference theory, the analytic framework used to derive the keypress metrics. *Refer to Table 3 for brain volume inclusion. #classified/n = the number of correctly classified participants out of the total number participants in a respective group; %classified = the percentage of correctly classified participants in a given group; #misclassified/n = the number of incorrectly classified participants out of the total number participants in a respective group; %classified = the percentage of incorrectly classified participants in a given group; N = the total sample size for a given kNN model.

The kNN ML algorithm was used to classify CTRL from MDD, CTRL from CD, and MDD from CD. Across all models (Fig 5A–5C), the average classification accuracy was 75% for CTRL, 29% for MDD, and 77% for CD. For the classification of CTRL and MDD, model accuracies were highest when brain volumes, radial dispersion, and demographics were included (87% for CTRL and 36% for MDD; Fig 5A). The same finding was observed for the classification of CTRL and CD (82% for both CTRL and CD; Fig 5B). As features were added to the model, accuracy improved. It is notable that radial dispersion, alone, produced classification accuracies as high as 76.1% and as high as 86.7% when demographic variables were also included. For the classification of MDD and CD, the accuracy for MDD was highest when only brain volumes and demographics were included (32%; Fig 5C) and the accuracy for CD was highest when all three variable types were included (91%, Fig 5C). In general, CTRL and CD were more accurately classified and MDD was often misclassified.

### *Post-hoc* results: Lack of relationships between impulsivity and keypressing

Of the 60 potential linear relationships between novelty seeking (an indicator of impulsivity) and keypress metrics, the only significant relationship was between mean h inc and novelty seeking for the MDD group (*p* = .034, *β* = 0.487, R^2^ = 0.237).

## Discussion

This study used an RPT-based operant keypress task and brain morphometry to quantify brain-behavior relationships and assess how well statistical interaction, or ML-related variable fusion, of keypress metrics and brain structure could discriminate between MDD, CD, and CTRL participants. Persons with MDD and CD are known to exhibit alterations in reward/aversion behaviors [19, 27–29], but differences in brain structure and other imaging metrics may lack consistency in the literature [8]. This study yielded three primary findings. First, CTRL, MDD, and CD participants had distinct linear relationships between brain structure and keypress metrics, with only 7.8–14% of associations overlapping between any two groups. Second, the three groups had statistically distinct slopes as demonstrated with multiple heatmap frameworks. Third, a ML approach, using early data fusion, could segregate CD from CTRL and MDD participants, but could not segregate MDD from CTRL or CD. These findings, using multiple discrimination frameworks, argue that variable interactions, and/or their fusion, may improve the classification of mental health conditions in lieu of standard approaches that do not evaluate sets of interactions or variable fusion.

### Distinct brain structure-behavior relationships point to biopsychological differences between CTRL, MDD, and CD

Brain volumes were regressed against keypress metrics to uncover distinct structure-behavior relationships in CTRL, MDD, and CD participants. Normalized brain volumes have been associated with reward/aversion keypress behavior in healthy volunteers [36]. Changes in keypress behavior and brain structure have also been observed in those with substance use problems and those with depression [36, 63–67], but differences in the *relationships* between brain structure and keypress behavior have not yet been established.

We observed that only *four* of the 102 structure-behavior relationships overlapped (with slope in the same direction) between any two groups (Table 1 and Fig 2). This observation suggests that the relationship of reward/aversion behavior and brain structure may be distinct between the studied CTRL, MDD, and CD participants. Furthermore, the pattern of keypress metrics showing significant regressions with brain structure were distinct by group. For instance, only CTRL exhibited regressions with negative turning point–a keypress metric that describes how much aversion toward an event/object an individual must overcome to avoid that goal-object. CTRL also lacked regressions against radial distance, whereas MDD and CD exhibited them. Radial distance is a keypress metric quantifying conflict versus indifference in judgments (e.g., having both low rewarding and low aversion valuations toward something; see S1 Appendix).

The three groups also showed distinct brain structures involved with these structure-behavior relationships: a) for CTRL, left cerebral white matter, right/left putamen, and right hippocampus, b) for MDD, right exterior cerebellum, right cerebellar cortex, and left pallidum, and c) for CD, right/left cerebral cortex. These brain structures, along with all structures listed in Table 1, have been implicated in reward and aversion brain processes, consistent with the general literature [66–86]. Uniquely, the structure-behavior relationships observed in this study involved a broad array of interpretable metrics around reward/aversion judgment, offering a more nuanced framework for assessing biopsychological differences between CTRL, MDD, and CD participants. To apply these findings at an individual level, a likelihood score of group membership could be generated by calculating the distance between *β* terms from each structure-behavior regression. Together, these observations support the use of variable interactions for discriminating psychiatric conditions [8].

### Quantitative differences between brain structure-behavior regression slopes validate distinctiveness

Assessment of structure-behavior slopes (*β*) revealed significant differences in the valence and intensity of the *β* terms, as well as differences in the direction (+/-) of the *β* terms (Fig 4 and Table 2). These observations suggest that interaction patterns using standard linear regression can be used to identify differences in structure-behavior relationships, thereby offering a quantitative method to discriminate between CTRL, MDD, and CD. The lack of relationships between novelty seeking (an indicator of impulsivity) and keypressing suggests that the relationships between behavior and brain structure were not driven by impulse control. The unique patterns observed from structure-behavior regression analysis 1) suggest there are major biopsychological differences between CTRL, MDD, and CD and 2) offer a potential framework to discriminate between these three groups. These methods can be extended to other psychiatric disorders to aide psychiatric evaluation.

### ML classified CTRL and CD, but not MDD

This study used a kNN ML approach that included variable fusion for diagnostic categorization. Variables used for kNN classification included the radial dispersion keypress metric and nine brain volumes (Table 3). Radial dispersion reflects the variance in how much an individual goes between having conflicting (i.e., wanting and not wanting) and indifferent (neither wanting or not wanting) approach/avoidance behavior. It is one of two RPT metrics that define how broad an individual’s preference portfolio is, which tends to be broader in healthy individuals. The nine brain volumes have been shown to vary in those with substance use problems and those with major depression [66–85].

The features described above, along with demographic variables (gender, age, ethnicity, and years of education), were inputs for kNN. Overall, kNN performed well for CTRL and CD classification (58–91% accuracy) but not for MDD classification (16–36% accuracy). This could be attributed, in part, to two factors: 1) the small sample size (n = 25) for the MDD group may not yield enough data points to develop a clear classification cluster, and 2) MDD is characterized as a heterogenous disorder [9, 10] with potentially 56 subtypes based on having any five of the eight neurovegetative symptoms over two weeks for a formal diagnosis [16]. This potential variance across MDD participants may have overpowered the small sample size. This observation is in alignment with a recent review article highlighting the lack of consistent neuroimaging findings across studies of MDD, owing to its heterogeneity and suggesting that studies should move past simple univariate analysis to incorporate quantitative, theory-driven computational approaches and multivariate prediction [8], both of which have been demonstrated in this study.

### Limitations

There are several limitations that should be addressed. The small sample size, especially for MDD, represents a limitation to extrapolating findings to the general population [87]. On the other hand, remarkably few neuroimaging studies use psychological primitives that have been tested for Feynman lawfulness [88], like the RPT-based keypress metrics used in this study [23, 26], which, by their mathematical discreteness, increase the reliability of findings. A second limitation regards the brain volume segmentation used. In the present study, larger brain volumes were segmented (e.g., posterior parietal cortex) which may result in smaller parcellation units with known functional differences being averaged together and thus masked. However, this could be seen as an advantage as smaller brain regions are often difficult to segment in a reproducible manner (e.g., substantia nigra vs. ventral tegmentum). Future work should incorporate segmentation of both larger brain regions as well as smaller, more targeted regions. A well-validated, anatomist-supervised approach to brain segmentation was also used in this study which produces greater variance in measures than automated approaches like Freesurfer (e.g., [36]), suggesting the findings herein may be underpowered relative to use of automated segmentation techniques.

## Conclusions

This study integrated computational behavior measures of reward/aversion (via a keypress task and RPT analysis) with structural brain imaging to study 1) group differences in structure-behavior regressions, 2) qualitative and quantitative heatmap assessment of structure-behavior associations, and 3) classification of CTRL, MDD, and CD participants using ML. The three groups exhibited distinct relationships between brain structure and reward/aversion behavior–pointing to clear biopsychological differences. These distinctions were better observed using standard linear regression and qualitative and quantitative heatmap assessment, as compared to the chosen ML approach. Our findings suggest that these disorders can, in part, be categorized by distinct interactions between reward/aversion judgment and the brain regions involved with judgment. The observed interaction patterns may be useful for improving psychiatric evaluation accuracy, especially if paired with next-generation, broadly available MRIs [89, 90]. Given hypotheses that reward/aversion judgment may show unique abnormalities across other psychiatric diagnoses [14, 15], the methods described herein might be relevant for broader classification of psychiatric illness.

## Supporting information

S1 Appendix Extended methods. (DOCX)

S2 Appendix Dataset. (XLSX)

S3 Appendix Members of the Massachusetts general hospital phenotype genotype project in addiction and mood disorders. (XLSX)

S1 Fig Structure-behavior regression plots. Results were plotted using regression results from Table 1 where covariates were included.
(DOCX)

S2 Fig Overlapping structure-behavior regression plots without covariate inclusion. (DOCX)

S1 Table Demographic information. (DOCX)

S2 Table Demographic differences by group. (DOCX)

S3 Table Brain volume abbreviations and descriptions. (DOCX)

S4 Table Brain volume differences by demographic variables. (DOCX)

S5 Table Overlapping brain regions using structure-behavior regression relationships (with covariates) presented in Table 1. Overlapping regressions presented in Fig 2 with inclusion of covariates were excluded.
(DOCX)

S6 Table Structure-behavior regression results without covariate inclusion. (DOCX)

S7 Table Overlapping brain regions using structure-behavior regression relationships (without covariates) presented in S6 Table. Overlapping regressions presented in S2 Fig without inclusion of covariates were excluded.
(DOCX)

## References

[1] Davis L, Uezato A, Newell JM, Frazier E (2008): Major depression and comorbid substance use disorders. *Curr Opin Psychiatry* 21: 14–18. doi: 10.1097/YCO.0b013e3282f32408 18281835

[2] Quello SB, Brady KT, Sonne SC (2005): Mood Disorders and Substance Use Disorder: A Complex Comorbidity. *Science & Practice Perspectives* 3: 13–21. doi: 10.1151/spp053113 18552741 PMC2851027

[3] Dickey B, Normand SLT, Weiss RD, Drake RE, Azeni H (2002): Medical morbidity, mental illness, and substance use disorders. *Psychiatric Services* 53: 861–867. doi: 10.1176/appi.ps.53.7.861 12096170

[4] Torrens M, Rossi PC, Martinez-Riera R, Martinez-Sanvisens D, Bulbena A (2012): Psychiatric Co-Morbidity and Substance Use Disorders: Treatment in Parallel Systems or in One Integrated System? *Substance Use and Misuse* 47: 1005–1014. doi: 10.3109/10826084.2012.663296 22676568

[5] Thaipisuttikul P, Ittasakul P, Waleeprakhon P, Wisajun P, Jullagate S (2014): Psychiatric comorbidities in patients with major depressive disorder. *Neuropsychiatric Disease and Treatment* 10: 2097–2103. doi: 10.2147/NDT.S72026 25419132 PMC4235207

[6] Filatova E v., Shadrina MI, Slominsky PA (2021): Major Depression: One Brain, One Disease, One Set of Intertwined Processes. *Cells* 10: 1283. doi: 10.3390/cells10061283 34064233 PMC8224372

[7] Carroll KM (2021): The Profound Heterogeneity of Substance Use Disorders: Implications for Treatment Development. *Current Directions in Psychological Sciences* 30: 358–364. doi: 10.1177/09637214211026984 34483503 PMC8415637

[8] Winter NR, Leenings R, Ernsting J, Sarink K, Fisch L, Emden D, et al. (2021): Quantifying deviations of brain structure and function in major depressive disorder across neuroimaging modalities. *JAMA Psychiatry* 79: 879–888. 10.1001/jamapsychiatry.2022.1780 PMC9330277 35895072

[9] Athira KV, Bandopadhyay S, Samudrala PK, Naidu VGM, Lahkar M, Chakravarty S (2019): An Overview of the Heterogeneity of Major Depressive Disorder: Current Knowledge and Future Prospective. *Current Neuropharmacology* 18: 168–187. 10.2174/1570159X17666191001142934 PMC7327947 31573890

[10] Goldberg D (2011): The heterogeneity of “major depression.” *World Psychiatry* 10: 226–228. 21991283 10.1002/j.2051-5545.2011.tb00061.x PMC3188778

[11] Worley MJ, Trim RS, Roesch SC, Mrnak-Meyer J, Tate SR, Brown SA (2012): Comorbid Depression and Substance Use Disorder: Longitudinal Associations Between Symptoms in a Controlled Trial. *Journal of Substance Abuse Treatment* 43: 291–302. doi: 10.1016/j.jsat.2011.12.010 22406052 PMC3382030

[12] Dakwar E, Nunes E v., Bisaga A, Carpenter KC, Mariani JP, Sullivan MA, et al. (2011): A Comparison of Independent Depression and Substance-Induced Depression in Cannabis, Cocaine, and Opioid Dependent Treatment Seekers. *The American Journal on Addictions* 20: 441–446. doi: 10.1111/j.1521-0391.2011.00148.x 21838843 PMC3600842

[13] Glantz MD, Conway KP, Colliver JD (2005): Drug Abuse Heterogeneity and the Search for Subtypes. *Epidemiology of Drug Abuse*. Springer, Boston, MA, pp 15–27.

[14] Breiter HC, Gasic GP (2004): A General Circuitry Processing Reward/Aversion Information and Its Implications for Neuropsychiatric Illness. In: Boston Review, editor. Gazzaniga M. S *(Ed*.*)*, *The Cognitive Neurosciences*. pp 1043–1065.

[15] Breiter HC, Gasic GP, Makris N (2006): Imaging the Neural Systems for Motivated Behavior and Their Dysfunction in Neuropsychiatric Illness. Deisboeck T.S., Kresh J.Y. *(Eds)* *Complex Systems Science in Biomedicine*. *Topics in Biomedical Engineering International Book Series*. Springer, Boston, MA, pp 763–810.

[16] Pizzagalli DA, Iosifescu D, Hallett LA, Ratner KG, Fava M (2008): Reduced Hedonic Capacity in Major Depressive Disorder: Evidence from a Probabilistic Reward Task. *Journal of Psychiatric Research* 43: 76–87. doi: 10.1016/j.jpsychires.2008.03.001 18433774 PMC2637997

[17] Insel T, Cuthbert B, Garvey M, Heinssen R, Pine DS, Quinn K, et al. (2010): Research domain criteria (RDoC): toward a new classification framework for research on mental disorders. *Am J Psychiatry* 167: 748–751. doi: 10.1176/appi.ajp.2010.09091379 20595427

[18] White NM (1986): Control of sensorimotor function by dopaminergic nigrostriatal neurons: influence on eating and drinking. *Neurosci Biobehav Rev* 10: 15–36. doi: 10.1016/0149-7634(86)90030-8 3010199

[19] Aharon I, Etcoff N, Ariely D, Chabris CF, O’Connor E, Breiter HC (2001): Beautiful faces have variable reward value: fMRI and behavioral evidence. *Neuron* 32: 537–551. doi: 10.1016/s0896-6273(01)00491-3 11709163

[20] Strauss MM, Makris N, Aharon I, Vangel MG, Goodman J, Kennedy DN, et al. (2005): fMRI of sensitization to angry faces. *Neuroimage* 26: 389–413. doi: 10.1016/j.neuroimage.2005.01.053 15907298

[21] Perlis RH, Holt DJ, Smoller JW, Blood AJ, Lee S, Kim BW, et al. (2008): Association of a polymorphism near CREB1 with differential aversion processing in the insula of healthy participants. *Archives of General Psychiatry* 65: 882–892. doi: 10.1001/archgenpsychiatry.2008.3 18678793 PMC3782742

[22] Gasic GP, Smoller JW, Perlis RH, Sun M, Lee S, Kim BW, et al. (2009): BDNF, relative preference, and reward circuitry responses to emotional communication. *American Journal of Medical Genetics*, *Part B*: *Neuropsychiatric Genetics* 150: 762–781. doi: 10.1002/ajmg.b.30944 19388013 PMC7891456

[23] Kim BW, Kennedy DN, Lehár J, Lee MJ, Blood AJ, Lee S, et al. (2010): Recurrent, robust and scalable patterns underlie human approach and avoidance. *PLoS ONE* 5: e10613. doi: 10.1371/journal.pone.0010613 20532247 PMC2879576

[24] Lee S, Lee MJ, Kim BW, Gilman JM, Kuster JK, Blood AJ, et al. (2015): The commonality of loss aversion across procedures and stimuli. *PLoS ONE* 10: e0135216. doi: 10.1371/journal.pone.0135216 26394306 PMC4579072

[25] Viswanathan V, Lee S, Gilman JM, Kim BW, Lee N, Chamberlain L, et al. (2015): Age-related striatal BOLD changes without changes in behavioral loss aversion. *Frontiers in Human Neuroscience* 9: 176. doi: 10.3389/fnhum.2015.00176 25983682 PMC4415398

[26] Livengood SL, Sheppard JP, Kim BW, Malthouse EC, Bourne JE, Barlow AE, et al. (2017): Keypress-Based Musical Preference Is Both Individual and Lawful. *Frontiers in Neuroscience* 11: 136. doi: 10.3389/fnins.2017.00136 28512395 PMC5412065

[27] Halahakoon DC, Kieslich K, O’Driscoll C, Nair A, Lewis G, Roiser JP (2020): Reward-Processing Behavior in Depressed Participants Relative to Healthy Volunteers: A Systematic Review and Meta-analysis. *JAMA Psychiatry* 77: 1286–1295. doi: 10.1001/jamapsychiatry.2020.2139 32725180 PMC7391183

[28] Volkow ND, Wang GJ, Fowler JS, Tomasi D, Telang F, Baler R (2010): Addiction: Decreased reward sensitivity and increased expectation sensitivity conspire to overwhelm the brain’s control circuit. *BioEssays* 32: 748–755. doi: 10.1002/bies.201000042 20730946 PMC2948245

[29] Breiter HC, Gollub RL, Weisskoff RM, Kennedy DN, Makris N, Berke JD, et al. (1997): Acute effects of cocaine on human brain activity and emotion. *Neuron* 19: 591–611. doi: 10.1016/s0896-6273(00)80374-8 9331351

[30] Paulus MP, Yu AJ (2012): Emotion and decision-making: affect-driven belief systems in anxiety and depression. *Trends in Cognitive Sciences* 16: 476–483. doi: 10.1016/j.tics.2012.07.009 22898207 PMC3446252

[31] Keren H, O’Callaghan G, Vidal-Ribas P, Buzzell GA, Brotman MA, Leibenluft E, et al. (2018): Reward Processing in Depression: A Conceptual and Meta-Analytic Review Across fMRI and EEG Studies. *The American Journal of Psychiatry* 175: 1111–1120. doi: 10.1176/appi.ajp.2018.17101124 29921146 PMC6345602

[32] Bai T, Zu M, Chen Y, Xie W, Cai C, Wei Q, et al. (2018): Decreased Connection Between Reward Systems and Paralimbic Cortex in Depressive Patients. *Frontiers in Neuroscience* 12: 462. doi: 10.3389/fnins.2018.00462 30038557 PMC6046444

[33] Breiter HC, Rosen BR (1999): Functional magnetic resonance imaging of brain reward circuitry in the human. *Ann N Y Acad Sci* 877: 523–547. doi: 10.1111/j.1749-6632.1999.tb09287.x 10415669

[34] Hayes DJ, Northoff G (2011): Identifying a Network of Brain Regions Involved in Aversion-Related Processing: A Cross-Species Translational Investigation. *Frontiers in Integrative Neuroscience* 5: 49. doi: 10.3389/fnint.2011.00049 22102836 PMC3215229

[35] Viswanathan V, Sheppard JP, Kim BW, Plantz CL, Ying H, Lee MJ, et al. (2017): A Quantitative Relationship between Signal Detection in Attention and Approach/Avoidance Behavior. *Frontiers in Psychology* 8: 122. doi: 10.3389/fpsyg.2017.00122 28270776 PMC5318395

[36] Makris N, Gasic GP, Kennedy DN, Hodge SM, Kaiser JR, Lee MJ, et al. (2008): Cortical Thickness Abnormalities in Cocaine Addiction-A Reflection of Both Drug Use and a Pre-existing Disposition to Drug Abuse? *Neuron* 60: 174–188. doi: 10.1016/j.neuron.2008.08.011 18940597 PMC3772717

[37] Blood AJ, Iosifescu D v., Makris N, Perlis RH, Kennedy DN, Dougherty DD, et al. (2010): Microstructural Abnormalities in Subcortical Reward Circuitry of Subjects with Major Depressive Disorder. *PLoS ONE* 5: e13945. doi: 10.1371/journal.pone.0013945 21124764 PMC2993928

[38] Makris N, Gasic GP, Seidman LJ, Goldstein JM, Gastfriend DR, Elman I, et al. (2004): Decreased absolute amygdala volume in cocaine addicts. *Neuron* 44: 729–740. doi: 10.1016/j.neuron.2004.10.027 15541319

[39] Fischl B, Salat DH, van der Kouwe AJW, Makris N, Ségonne F, Quinn BT, et al. (2004): Sequence-independent segmentation of magnetic resonance images. *Neuroimage* 23: S69–S84. doi: 10.1016/j.neuroimage.2004.07.016 15501102

[40] Fischl B, Salat DH, Busa E, Albert M, Dieterich M, Haselgrove C, et al. (2002): Whole brain segmentation: automated labeling of neuroanatomical structures in the human brain. *Neuron* 33: 341–355. doi: 10.1016/s0896-6273(02)00569-x 11832223

[41] Seidman LJ, Faraone S v, Goldstein JM, Kremen WS, Horton NJ, Makris N, et al. (2002): Left Hippocampal Volume as a Vulnerability Indicator for Schizophrenia: A Magnetic Resonance Imaging Morphometric Study of Nonpsychotic First-Degree Relatives. *Archives of General Psychiatry* 59: 839–849. doi: 10.1001/archpsyc.59.9.839 12215084

[42] Goldstein JM, Goodman JM, Seidman LJ, Kennedy DN, Makris N, Lee H, et al. (1999): Cortical Abnormalities in Schizophrenia Identified by Structural Magnetic Resonance Imaging. *Arch Gen Psychiatry* 56: 537–547. doi: 10.1001/archpsyc.56.6.537 10359468

[43] Seidman LJ, Faraone S v, Goldstein JM, Goodman JM, Kremen WS, Toomey R, et al. (1999): Thalamic and amygdala–hippocampal volume reductions in first-degree relatives of patients with schizophrenia: an MRI-based morphometric analysis. *Biological Psychiatry* 46: 941–954. doi: 10.1016/s0006-3223(99)00075-x 10509177

[44] Worth AJ, Makris N, Caviness VS, Kennedy DN (1997): Neuroanatomical Segmentation in MRI: Technological Objectives. *International Journal of Pattern Recognition and Artificial Intelligence* 11: 1161–1187.

[45] Makris N, Kaiser J, Haselgrove C, Seidman LJ, Biederman J, Boriel D, et al. (2006): Human cerebral cortex: A system for the integration of volume-and surface-based representations. *Neuroimage* 33: 139–153. doi: 10.1016/j.neuroimage.2006.04.220 16920366

[46] Caviness VS, Kennedy DN, Richelme C, Rademacher J, Filipek PA (1996): The human brain age 7–11 years: a volumetric analysis based on magnetic resonance images. *Cereb Cortex* 6: 726–736. doi: 10.1093/cercor/6.5.726 8921207

[47] Caviness VS, Meyer J, Makris N, Kennedy DN (1996): MRI-Based Topographic Parcellation of Human Neocortex: An Anatomically Specified Method with Estimate of Reliability. *J Cogn Neurosci* 8: 566–587. doi: 10.1162/jocn.1996.8.6.566 23961985

[48] Filipek PA, Richelme C, Kennedy DN, Caviness VS (1994): The young adult human brain: an MRI-based morphometric analysis. *Cereb Cortex* 4: 344–360. doi: 10.1093/cercor/4.4.344 7950308

[49] Breiter HC, Filipek PA, Kennedy DN, Baer L, Pitcher DA, Olivares MJ, et al. (1994): Retrocallosal White Matter Abnormalities in Patients With Obsessive-compulsive Disorder. *Archives of General Psychiatry* 51: 663–664. doi: 10.1001/archpsyc.1994.03950080075010 8042915

[50] StataCorp (2021): Stata Statistical Software: Release 17. College Station, TX: StataCorp LLC. College Station, TX: StataCorp LLC.

[51] R Core Team (2021): R: A language and environment for statistical computing. R Foundation for Statistical Computing, Vienna, Austria: https://www.r-project.org/. https://doi.org/https://www.r-project.org/

[52] Azcona EA, Kim B-W, Vike NL, Bari S, Lalvani S, Stefanopoulos L, et al. (2022): Discrete, recurrent, and scalable patterns in human judgement underlie affective picture ratings. *arXiv* arXiv:2203.06448. 10.48550/arxiv.2203.06448 PMC12055920 39644430

[53] Cook RD (1977): Detection of influential observations in linear regression. *Technometrics* 22: 494–508.

[54] Cook RD (1979): Influential observations in linear regression. *J Am Stat Assoc* 74: 169–174.

[55] Wilcoxon F (1945): Individual Comparisons by Ranking Methods. *Biometrics Bulletin* 1: 80–83.

[56] Spearman C (1904): The Proof and Measurement of Association between Two Things. *The American Journal of Psychiatry* 15: 72–101. 3322052

[57] Benjamini Y, Hochberg Y (1995): Controlling the False Discovery Rate: A Practical and Powerful Approach to Multiple Testing. *Journal of the Royal Statistical Society Series B (Methodological)* 57: 289–300.

[58] Acock AC (2014): *A Gentle Introduction to Stata*, 4th ed. College Station, TX: Stata Press.

[59] Dinno A (2015): Nonparametric pairwise multiple comparisons in independent groups using Dunn’s test. *Stata Journal* 15: 292–300.

[60] Fix E, Hodges JL (1951): Discriminatory Analysis, Nonparametric Discrimination: Consistency Properties. *International Statistical Review* 57: 238–247.

[61] Rencher AC, Christensen WF (2012): *Methods of Multivariate Analysis*, 3rd ed. John Wiley & Sons, Inc.

[62] Keller JM, Gray MR (1985): A Fuzzy K-Nearest Neighbor Algorithm. *IEEE Transactions on Systems*, *Man and Cybernetics* SMC-15: 580–585.

[63] Loijen A, Vrijsen JN, Egger JIM, Becker ES, Rinck M (2020): Biased approach-avoidance tendencies in psychopathology: A systematic review of their assessment and modification. *Clinical Psychology Review* 77: 101825. doi: 10.1016/j.cpr.2020.101825 32143108

[64] Nguyen D, Schumacher A, Erb S, Ito R (2015): Aberrant approach-avoidance conflict resolution following repeated cocaine pre-exposure. *Psychopharmacology (Berl)* 232: 3573–3583. doi: 10.1007/s00213-015-4006-y 26156635

[65] Struijs SY, Lamers F, Vroling MS, Roelofs K, Spinhoven P, Penninx BWJH (2017): Approach and avoidance tendencies in depression and anxiety disorders. *Psychiatry Research* 256: 475–481. doi: 10.1016/j.psychres.2017.07.010 28715782

[66] Dai L, Zhou H, Xu X, Zuo Z (2019): Brain structural and functional changes in patients with major depressive disorder: A literature review. *PeerJ* 7: e8170. doi: 10.7717/peerj.8170 31803543 PMC6886485

[67] Nestler EJ (2005): The Neurobiology of Cocaine Addiction. *Science & Practice Perspectives* 3: 4–10. doi: 10.1151/spp05314 18552739 PMC2851032

[68] Pandya M, Altinay M, Malone DA, Anand A (2012): Where in the Brain Is Depression? *Current Psychiatry Reports* 14: 634–642. doi: 10.1007/s11920-012-0322-7 23055003 PMC3619732

[69] Ayyash S, Davis AD, Alders GL, MacQueen G, Strother SC, Hassel S, et al. (2021): Exploring brain connectivity changes in major depressive disorder using functional-structural data fusion: A CAN-BIND-1 study. *Hum Brain Mapp* 42: 4940–4957. doi: 10.1002/hbm.25590 34296501 PMC8449113

[70] Ma L, Steinberg JL, Moeller FG, Johns SE, Narayana PA (2015): Effect of cocaine dependence on brain connections: Clinical implications. *Expert Rev Neurother* 15: 1307–1319. doi: 10.1586/14737175.2015.1103183 26512421 PMC4651809

[71] Jedema HP, Song X, Aizenstein HJ, Bonner AR, Stein EA, Yang Y, Bradberry CW (2021): Long-Term Cocaine Self-administration Produces Structural Brain Changes That Correlate With Altered Cognition. *Biol Psychiatry* 89: 376–385. doi: 10.1016/j.biopsych.2020.08.008 33012519 PMC7855373

[72] Depping MS, Schmitgen MM, Kubera KM, Wolf RC (2018): Cerebellar Contributions to Major Depression. *Frontiers in Psychiatry* 9: 634. doi: 10.3389/fpsyt.2018.00634 30555360 PMC6281716

[73] Phillips JR, Hewedi DH, Eissa AM, Moustafa AA (2015): The Cerebellum and Psychiatric Disorders. *Frontiers in Public Health* 3: 66. doi: 10.3389/fpubh.2015.00066 26000269 PMC4419550

[74] Miquel M, Gil-Miravet I, Guarque-Chabrera J (2020): The Cerebellum on Cocaine. *Frontiers in Systems Neuroscience* 14: 586574. doi: 10.3389/fnsys.2020.586574 33192350 PMC7641605

[75] Vazquez-Sanroman D, Leto K, Cerezo-Garcia M, Carbo-Gas M, Sanchis-Segura C, Carulli D, et al. (2015): The cerebellum on cocaine: plasticity and metaplasticity. *Addiction biology* 20: 941–955. doi: 10.1111/adb.12223 25619460

[76] Ide JS, Zhang S, Hu S, Sinha R, Mazure CM, Li C shan R (2014): Cerebral gray matter volumes and low-frequency fluctuation of BOLD signals in cocaine dependence: Duration of use and gender difference. *Drug and Alcohol Dependence* 134: 51–62. doi: 10.1016/j.drugalcdep.2013.09.004 24090712 PMC3865077

[77] Brown EC, Clark DL, Hassel S, MacQueen G, Ramasubbu R (2017): Thalamocortical connectivity in major depressive disorder. *J Affect Disord* 217: 125–131. doi: 10.1016/j.jad.2017.04.004 28407555

[78] Zhang S, Hu S, Sinha R, Potenza MN, Malison RT, Li C shan R (2016): Cocaine dependence and thalamic functional connectivity: a multivariate pattern analysis. *NeuroImage Clinical* 12: 348–358. doi: 10.1016/j.nicl.2016.08.006 27556009 PMC4986538

[79] Han KM, Kim D, Sim Y, Kang J, Kim A, Won E, et al. (2017): Alterations in the brainstem volume of patients with major depressive disorder and their relationship with antidepressant treatment. *J Affect Disord* 208: 68–75. doi: 10.1016/j.jad.2016.08.066 27750062

[80] Geisler S, Marinelli M, DeGarmo B, Becker ML, Freiman AJ, Beales M, et al. (2007): Prominent Activation of Brainstem and Pallidal Afferents of the Ventral Tegmental Area by Cocaine. *Neuropsychopharmacology* 33: 2688–2700. doi: 10.1038/sj.npp.1301650 18094667 PMC2978288

[81] Ancelin ML, Carrière I, Artero S, Maller J, Meslin C, Ritchie K, et al. (2019): Lifetime major depression and grey-matter volume. *Journal of Psychiatry & Neuroscience* 44: 45–53. doi: 10.1503/jpn.180026 30565905 PMC6306287

[82] Sacchet MD, Livermore EE, Iglesias JE, Glover GH, Gotlib IH (2015): Subcortical volumes differentiate Major Depressive Disorder, Bipolar Disorder, and remitted Major Depressive Disorder. *Journal of Psychiatric Research* 68: 91–98. doi: 10.1016/j.jpsychires.2015.06.002 26228406 PMC11887997

[83] Bittencourt AML, Bampi VF, Sommer RC, Schaker V, Juruena MFP, Soder RB, et al. (2021): Cortical thickness and subcortical volume abnormalities in male crack-cocaine users. *Psychiatry Research*: *Neuroimaging* 310: 111232. doi: 10.1016/j.pscychresns.2020.111232 33621927

[84] Zhang S, Li CSR (2018): Ventral striatal dysfunction in cocaine dependence–difference mapping for subregional resting state functional connectivity. *Translational Psychiatry* 8: 1–11. 29915214 10.1038/s41398-018-0164-0 PMC6006289

[85] Zhang S, Zhornitsky S, Le TM, Li CSR (2019): Hypothalamic Responses to Cocaine and Food Cues in Individuals with Cocaine Dependence. *International Journal of Neuropsychopharmacology* 22: 754–764. doi: 10.1093/ijnp/pyz044 31420667 PMC6929672

[86] Zhang FF, Peng W, Sweeney JA, Jia ZY, Gong QY (2018): Brain structure alterations in depression: Psychoradiological evidence. *CNS Neurosci Ther* 24: 994–1003. doi: 10.1111/cns.12835 29508560 PMC6489983

[87] Marek S, Tervo-Clemmens B, Calabro FJ, Montez DF, Kay BP, Hatoum AS, et al. (2022): Reproducible brain-wide association studies require thousands of individuals. *Nature* 603: 654–660. doi: 10.1038/s41586-022-04492-9 35296861 PMC8991999

[88] Feynman RP (1965): *The Character of Physical Law*. Cambridge: The British Broadcasting Corporation.

[89] Geethanath S, Vaughan JT (2019): Accessible magnetic resonance imaging: A review. *J Magn Reson Imaging* 49: e65–e77. doi: 10.1002/jmri.26638 30637891

[90] Shen FX, Wolf SM, Bhavnani S, Deoni S, Elison JT, Fair D, et al. (2021): Emerging ethical issue10.1016/j.neuroimage.2021.118210 PMC8382487 34062266

