# Supplementary material for: Characterizing major depressive disorder and substance use disorder using heatmaps and variable interactions: The utility of operant behavior and brain structure relationships
Source: PLoS One. 2024 Mar 11;19(3):e0299528. doi: 10.1371/journal.pone.0299528 (PMC10927130; doi:10.1371/journal.pone.0299528)
Supplement: S1 Appendix — (DOCX) [file pone.0299528.s001.docx]

**S1 APPENDIX**

**METHODS: Participant recruitment**

Subjects were participants in the Massachusetts General Hospital (MGH) Phenotype Genotype Project (PGP) in Addiction and Mood disorders, an interdisciplinary study sponsored by the Office of National Drug Control Policy (ONDCP) and designed to identify phenotype-genotype relationships in addictions and mood disorders using multimodal assessments, including behavioral clinical-diagnostic, neuroimaging, and genetic characterization. Participants were recruited by direct advertisement and clinical referrals, with attention to recruiting healthy control subjects who matched demographics with the major depressive disorder (MDD) and cocaine dependent (CD) participants. All participants signed consent following the approval of the MGH Institutional Review Board. Seventy-seven healthy control (CTRL) subjects, 47 MDD, and 120 CD participants were scanned with 10 imaging sequences including three MPRAGE sequences for structural MRI. Participants were excluded based on the following: 1) currently suicidal or at risk for suicide in the judgment of the investigator; 2) pregnant women; 3) carrying a medical device incompatible with MRI (e.g., metal implants such as surgical clips or pacemakers) or significant claustrophobia or weight that would make MRI unfeasible; 4) serious medical illness including a known history of HIV-1+status; 5) subjects with insulin dependent diabetes mellitus (IDDM) or subjects with noninsulin dependent diabetes mellitus (NIDDM) and abnormal Hemoglobin A1C; 6) severe respiratory compromise; 7) history of head trauma with neurological sequelae, including multiple concussions and/or history of stroke; 8) history of seizure disorder, delirium, dementia, or mental disorders due to general medical conditions; 9) clinical or laboratory evidence of uncontrolled hypothyroidism or hyperthyroidism; and 10) subjects which, in the opinion of the Management Group running the PGP, were not able to participate safely in this study. In addition, subjects in the larger PGP study were screened for Hepatitis C (by Hepatitis C+ titer); no subjects included in this study tested positive for Hepatitis C.

Eligible participants were between 18-65 years old without any major medical illness known to influence brain structure or function, including neurologic disease, HIV, or Hepatitis C, as determined by assay. Female participants were specifically tested during the mid-follicular phase of their menstrual cycle based on timeline follow-back of menstrual history over three months and confirmed with a urine hormonal assay at the time of testing. All participants had normal or corrected-to-normal vision. All subjects underwent a screening visit during which demographic data were collected, and the Structured Clinical Interviews for DSM-IV (SCID) for alcohol, drug dependence, and screen for other Axis I psychiatric disorders was administered. Additional measures included the Anger Attacks Questionnaire, Edinburgh Handedness Questionnaire, the Hamilton Rating Scale for Depression (HAM-D), the Inventory of Depressive Symptomatology (IDDRS), the MGH Cognitive and Physical Functioning Questionnaire, the short form 36-item Health Survey (SF-36), the Symptom Questionnaire (SQ), the Temperament and Character Inventory (TCI), the Wechsler Abbreviated Scale of Intelligence (WASI), and blood and urine samples. A physical examination and an inventory of physical symptoms, complaints, medication use, and MRI compatibility were also documented. Makris *et al.* 2008 and Blood *et al.* have previously described the recruitment methods mentioned herein (1,2).

*Healthy control (CTRL) recruitment*

Healthy CTRL participants were free of lifetime and current Axis I psychiatric disorders including cocaine, alcohol, or nicotine dependence, serious medical illness, or history of head trauma. They also did not meet criteria for alcohol or nicotine abuse. CTRL participants were between the ages of 19 and 52 years (32.35 ± 9.94 years) and had between 12-25 years of education (16 ± 2.68 years), with no significant differences between men and women for educational history (Wilcoxon Rank Sum test *p*>0.05). Five CTRL participants identified as Black/African American, six as Asian, and 29 as White; 20 were female and 20 were male; 35 identified as right-handed and five identified as left-handed.

*Cocaine dependent (CD) participant recruitment and demographics*

For CD participants, the American Society of Addiction Medicine Patient Placement Criteria 2-Revised (ASAM-PPC2R) was administered. Substance use measures came from the ASAM-PPC2R and included: 1) retrospective reports of years of cocaine use and alcohol use, 2) the number of days of cocaine and/or alcohol use in the past 30 days prior to study, and 3) the amount of money spent on substances in the past 30 days (Supplemental Table 1C). Through the ASAM-PPC2R, the number of days of use over the 30 days before study were also recorded for nicotine, sedatives, cannabis, opioids, and other stimulants. All users rated cocaine as their predominant substance, although 18 of 20 subjects also used nicotine, and 16 of 20 used alcohol (Supplemental Table 1C).

CD participants were between the ages of 21 and 57 years (42.76 ± 7.94 years) and had between 10-18 years of education (13.24 ± 1.90 years), with no significant differences between men and women for both age and educational history (Wilcoxon Rank Sum test *p*>0.05). 25 CD participants identified as Black/African American, one as Asian, 19 as White, and one did not report ethnicity; 10 were female and 36 were male; 43 identified as right-handed and three identified as left-handed. CD participants met criteria for cocaine dependence by SCID but were free of other Axis I psychiatric disorders with the exception of depression; they further had no serious medical illness, no history of head trauma, and were not on psychotropic medication. By ASAM-PPC2R, CD participants all rated cocaine as their primary substance of use, above alcohol and nicotine.

*Major depressive disorder (MDD) participant recruitment and demographics*

25 of the 47 subjects meeting DSM-IV criteria for Major Depressive Disorder (MDD) diagnosed by physician-administered Structured Clinical Interview for DSM-IV Axis I Disorders - Patient Edition [SCID-P [59]] survived data exclusion. These participants were between the ages of 20 and 56 (37.76 $\pm$ 11.93) and had between 7-20 years of education (14.4 $\pm$ 3.45) with no significant differences between men and women for both age and educational history (Wilcoxon Rank Sum test *p*>0.05). Three MDD participants identified as Black/African American, one as Asian, and 21 as White; 12 were female and 12 were male; 24 identified as right-handed and one identified as left-handed All subjects were drawn from a larger study evaluating cocaine addicted, depressed, and control subjects [The Massachusetts General Hospital (MGH) Phenotype Genotype Project in Addiction and Depression (PGP; http://pgp.mgh.harvard.edu)].

MDD subjects were excluded if they met DSM-IV criteria for primary psychotic disorders, bipolar disorder, eating disorders, substance abuse disorders, generalized anxiety disorder, panic disorder, PTSD, or OCD by SCID interview (current or lifetime); healthy volunteers were excluded if they met DSM-IV criteria for any Axis I psychiatric disorder by SCID interview.

**Note**: Given the low number of participants identifying as Asian across all three groups (CTRL, MDD, and CD; eight total), these participants were excluded from analyses of ethnicity by group (see Supplemental Table 3A).

**METHODS: Statistical Analysis for Covariates**

Demographic variables (age, gender, ethnicity, handedness, and years of education) were analyzed for differences across groups (CTRL, MDD, CD) using one of two statistical tests [chi-squared test or Kruskal Wallis (3); $\alpha$=0.05]. Statistical assessment indicated that gender, age, ethnicity, and years of education were significantly different across the three groups (*p*<0.05; Supplemental Table 2) and were thus used as covariates in further statistical analysis.

**METHODS: Keypress task**

Reward measures were collected using a dual keypress procedure with model vs. non-model faces (4,5). The keypress task examined the reward valuation connected to observing each of the faces, which over the ensemble of faces defined a subject’s relative preferences for these stimuli (6,7). It followed procedures reported previously which used a stimulus set constituted from men and women who either made a living as models (i.e., model males or females, or MM and MF), or did not (i.e., non-model males or females, or NMM and NMF) (4). Each category of face characterized by gender and modeling experience was defined as a psychological ensemble. In this study, the picture stimuli consisted of non-famous, model and non-model human faces (20 MM, 20 MF, 20 NMM, and 20 NMF) for a total of 80 faces. Pictures were selected from print media and digitized at 600 dpi in 8-bit grayscale, spatially downsampled, and cropped to fit in an oval window that was 310-350 pixels wide by 47- pixels high using Photoshop 4.0 (Adobe Systems).

Keypress procedures were implemented with MATLAB scripts on a computer. For this procedure, the four categories of faces with 20 identities for each category were considered as items in an economic bag of goods. The objective was to determine their relative preferences for the items in this bag of goods compared to the default position. Subjects were told that they would be exposed to a series of pictures that if not interfered with, would change every eight seconds (the default valuation of 6 seconds + 2 second decision block). However, if they wanted a picture to disappear faster, they could alternate pressing one set of keys (#3 and #4 on the button box), whereas if they wanted a picture to stay longer on the screen, they could alternate pressing other keys (#1 and #2 on the button box). Subjects had a choice to do nothing (default condition), increase viewing time, decrease viewing time, or a combination of the two responses. This resulted in four potential options: (a) approach (positive keypress), (b) avoid (negative keypress), (c) approach and avoid, or (d) indifference (no keypress). A “slider” was displayed left of each picture to indicate total viewing time. Subjects were informed that the task would last approximately 20 minutes, and that this length was independent of any behavioral guidelines for the task, as was their overall payment (i.e., $30 for the offline task if it was completed).

Results for each participant were plotted as raster plots of the number of keypresses made to increase picture viewing time, or to reduce picture viewing time (6,8,9). The dependent measure of interest was the amount of work in units of keypress that subjects exerted in response to the different categories of stimuli (i.e., the units in keypress that the subjects traded for viewing time). The keypress procedure quantified both the valence and the amount of the value that each item or face picture had relative to the default position of 6 seconds of viewing time. In such a psychological framework, individuals make judgments regarding the relative valence of the perceived stimulus in terms of its potential positive or negative effects, and judgments regarding the intensity of these effects (10–12). Their effort toggling the keypress reflects payment to approach or avoid what they perceive to be positive or negative effects. This relative preference task directly recruits reward/aversion circuitry (4,8,13–15), and might be altered in addicts given cocaine dependent subjects demonstrate a narrowed set of relative preferences along with reduced cognitive control/regulation of reward-based decision-making (16–18). This circuitry has also been observed altered in individuals with MDD (19–24).

As a symbolic framework around the keypress task (5), let $\left\{ x_{1}, x_{2}, x_{3}, \ldots x_{n} \right\}=S$ where $S$ is a set of items in a viewable item set. The relationship of key press effort to viewing time received was defined by a resistive function: $t_{n}= \sum_{n=1}^{N} t_{(n-1)}+ \frac{A-t_{n-1}}{J}$ , where $t_{n}$ equals the new time achieved via keypressing, $t_{n-1}$equals the time prior to a keypress, *A* equals Ø seconds for key presses reducing the viewing time and 14 seconds for key presses increasing the viewing time, and *J* was a scaling constant set to 40. The complete set of relative preferences for *S*, $U_{S}$ where $U_{S}= \sum_{s=0}^{S} \alpha_{S}t_{N}^{S}$ and $\alpha=f(A,J,N)$is potentially unique for each item in *S*. If the individual does nothing, the default is: $U_{S}= \sum_{s=0}^{S} \alpha_{S}t_{N}^{S}$ = 6 seconds x 80 items = 480 seconds. With the trading of effort for changes in time, $U_{S}$ defines each individual set of relative preferences as a set of deviations from the default position: ${|6-t_{N}|}_{S}$.

To normalize keypress behavior for potential differences in motor coordination or resiliency across groups, we used a measure of maximum keypress capacity/speed. This data was collected before undergoing the relative preference task, during a 30 second test session. Subjects were instructed to keypress “Z” or “X” alternately on a computer keyboard as fast as possible with their dominant hand. While keypressing, they viewed a blue bar on a gray background in the monitor indicating their keypress rate. The results of the keypress capacity/speed test used in this study were (i) number of keypresses over the mean view time of each category of model/non-model face (i.e., MF, NMF, MM, NMM), or (ii) mean speed over 30 seconds in Hz. A dimensionless index was produced by division of the mean number of absolute keypresses $(k_{+}-|k_{-}|)$ for each category of model/non-model face, by the maximum number of keypresses produced during the capacity/speed test for the time interval spent viewing each category of face. For quality assurance, we checked that it produced a result proportional to division of the mean keypress rate (in Hz) for each experimental condition by the mean speed (in Hz) during the capacity/speed test.

**METHODS: T1-weighted MRI parameter details**

Three sagittal 3D Magnetization-Prepared Rapid Acquisition Gradient Echo (MP-RAGE) T_1_-weighted sequences were acquired: TR = 2730 ms, TE = 3.31 ms, T1 = 1000 ms, flip angle = 7°, bandwidth = 195 Hz/pixel, FOV = 256x256 mm^2^, sampling matrix = 256x192 pixels, 128 contiguous 1.33 mm slices, averages = 3.

**METHODS: Image preprocessing, parcellation, and MRI-based segmentation**

Segmentation was performed with double blinding to group category and study hypotheses for all individuals performing data analysis. Neuroanatomic segmentation was performed using semi-automated intensity contour algorithms for external border definition and signal intensity histogram distributions for delineation of gray-white borders for cortex and subcortical structures as described extensively in prior publications (25–34). This technique allows for border definition as the midpoint between the peaks of the bimodal distribution for any given structure and its surrounding tissue (30). Segmentation of cortical and subcortical regions was performed on coronal images and divided the brain into gray matter and white matter regions. The cerebrum was segmented into its principal gray matter and white matter structures and total cerebral white matter. Specifically, the cortical ribbon was defined by two outlines, one external outline between the subarachnoid CSF and the cerebral cortex, and the other between the cerebral cortex and the underlying cerebral white matter (29,30). The total number of voxels in each cortical and subcortical region determined its volume. From a historical perspective, it is intriguing to note that the method of segmentation used herein was used as the gold-standard for validating the automated FreeSurfer segmentation and parcellation methodology upon its release (35,36).

**METHODS: Statistical analyses: Keypress feature extraction and descriptions**

This analysis used a feature space characterizing approach and avoidance judgments (6,7,9) that has been tightly linked to reward/aversion circuitry in the human brain (1,4,8,13–15). The keypress behavior to increase view time and the keypress behavior to decrease view time of each category of picture (MM, MF, NMM, NMF) were separated. Each participant’s keypressing behavior was be modeled over each of the four picture categories (MM, MF, NMM, NMF) to produce variables of keypress mean magnitude (***K***), variance or standard deviation ($\boldsymbol{\sigma}$), and uncertainty from the pattern of keypressing (Shannon entropy, ***H***). These variables were then plotted as ***KH***, ***K***$\boldsymbol{\sigma}$, and ***H^+^H^-^*** graphs. ***K^+^H^+^*** and ***K^+^***$\boldsymbol{\sigma}$***^+^*** are plotted separately from ***K^-^H^-^*** and ***K^-^***$\boldsymbol{\sigma}$***^-^****,* thereby calibrating approach/avoidance, ***K***, to the pattern of prior judgments, ***H***, and their variance, $\boldsymbol{\sigma}$. These three variables (***K****,* ***H****,* $\boldsymbol{\sigma}$) show consistent functions in their relationship, two of which resemble graphs observed in prospect theory (37) and another two of which resemble the graphs for the risk-reward function in portfolio theory (38). These values were plotted as curves (see Figure 1) in MATLAB using the library *polyfit* for each (6,7,9). The mean variables across category of picture were also incorporated into our analyses. Thus, (***K^+^,*** $\boldsymbol{K}$ ***^-^***), (***H^+^, H^-^***), and ($\boldsymbol{\sigma}$**^+^*,*** $\boldsymbol{\sigma}$***^-^***) were used in all analyses and are referred to as mean k inc (***K^+^***), mean k dec (***K^-^***), mean h inc (***H^+^***), mean h dec (***H^-^***), mean std inc ($\boldsymbol{\sigma}$**^+^**), and mean std inc ($\boldsymbol{\sigma}$**^-^**), accordingly.

An array of graph features was extracted from each curve that can be interpreted psychologically. The features used from these graphs are described below in psychological terms and organized relative to the curve from which they were extracted.

(1) The (***K, H***) curve, which consists of (***K^+^, H ^+^***) on the positive x-axis and (***K^-^, H^-^***) on the negative x-axis is analogous to the value function from Kahneman’s Prospect Theory (Kahneman 1979). The RPT features that can be extracted from this curve include *Risk Aversion, Loss Resilience, Loss Aversion,* and *Positive* and *Negative Offset.* Given only *Loss Resilience* and *Negative Offset* could be consistently computed for all subjects, only these two features from the (***K, H***) curves were used in subsequent analyses.

*Loss Resilience*: Loss resilience is defined as the absolute value of the ratio of the second derivative of the (***K^-^, H^-^***) curve to its first derivative, which also produces a curve. For prediction, we calculated *Loss Aversion* at ***K^-^*** = 1.5. Informally, *Loss Resilience* is the degree to which an individual prefers to lose a small defined amount in comparison to losing a greater amount with more uncertainty associated with this loss.

*Negative Offset*: *Negative Offset* is the value of ***K^-^*** when setting ***H^-^***= 0. It intuitively measures how much insurance an individual might need against bad outcomes. It mirrors the ante, but in the framework of potential losses.

(2) The (***K,*** $\boldsymbol{\sigma}$) curve, which consists of (***K^+^,*** $\boldsymbol{\sigma}$ ***^+^***) on the positive x-axis and (***K^-^,*** $\boldsymbol{\sigma}$ ***^-^***) on the negative x-axis is analogous to the mean-variance curve derived from portfolio theory (38). This allows comparison of preference magnitude (i.e., ***K***) with respect to variance in rewards and sanctions. The (***K,*** $\boldsymbol{\sigma}$) curve models the following question: Would an individual prefer a dollar with probability one, or value drawn from a normal distribution with mean of two and variance of two? The RPT features that are extracted from this curve include *Positive and Negative Apex, Positive and Negative Turning Point,* and *Positive and Negative Quadratic Area.* Given only *Negative Apex*, *Negative Turning Point*, and *Negative Quadratic Area* could be consistently computed from the (***K,*** $\boldsymbol{\sigma}$) curve for all subjects, only these three features were used in subsequent analyses.

*Negative Apex*: The *Negative Apex* is value of $\boldsymbol{\sigma}$ ***^-^*** where the derivative d $\boldsymbol{\sigma}$ -/d ***K^-^*** is equal to zero. Intuitively, this represents the maximum variance for avoidance behavior. Like with the *Positive Apex*, this transition point is important to consider for avoidance decisions in the context of Markowitz’s decision utility (38).

*Negative Turning Point*: The *Negative Turning Point* is the value of ***K^-^*** where the derivative d $\boldsymbol{\sigma}$ ***^-^***/d ***K^-^*** is equal to zero. Intuitively, this represents the rating intensity with maximum variance for approach behavior, potentially when an individual decides to avoid a goal-object.

*Negative Quadratic Area*: The *Negative Quadratic Area* is the area under the curve on the negative quadrant of the graph of (***K^-^,*** $\boldsymbol{\sigma}$ ***^-^***). Intuitively, this variable represents the relationship between ***K^-^*** and $\boldsymbol{\sigma}$ ***^-^*** and can be thought of as a quantity that measures the amount of overall value a person associates to a negative stimulus.

(3) The (***H^+^, H^-^***) curve allows the comparison of patterns in approach and avoidance judgments. The RPT features that are extracted from this curve include *Mean Polar Angle, Polar Angle Standard Deviation, Mean Radial Distance,* and *Radial Distance Standard Deviation.*

*Mean Polar Angle*: The *Mean Polar Angle* is the mean of the polar angles of the points in the (***H^+^, H^-^***) plane. Intuitively, this measures the mean ratio of entropies or patterns in approach to avoidance behavior.

*Polar Angle Standard Deviation (Polar Dispersion)*: This is the standard deviation of the polar angles of the points in the (***H^+^, H^-^***) plane. Intuitively, this measures the standard deviation in the patterns of approach to avoidance behavior. This variance represents the spread for positive preferences and negative preferences across a set of potential goal-objects and is one measure of the breadth of an individual’s (or group’s) preferences.

*Mean Radial Distance*: The *Mean Radial Distance* measures the mean of the distances of the data points in the (***H^+^, H^-^***) curve to the origin. Intuitively, this defines how individuals can have strong preferences (i.e., biases) for the same thing, reflecting *conflict*, or having low preferences for something, reflecting *indifference*. This gets at the consistency or compatibility of approach and avoidance, and how you can both like and dislike something, or be indifferent to both its positive and negative features.

*Radial Distance Standard Deviation (Radial Dispersion)*: The *Radial Distance Standard Deviation* measures the standard deviation of the distances of the data points in the (***H^+^, H^-^***) plane to the origin. Intuitively, this measures how the points in the HH plane vary with regard to radial distance from the origin. The variance in this radial distance will reflect how much an individual goes between having *conflicting* preferences and having *indifferent* ones.

**METHODS: Statistical analyses: kNN**

kNN is a supervised machine learning approach commonly used to solve classification problems (i.e., predicting the correct grouping using the data provided). kNN works by first specifying the number of nearest neighbors (***k***) you want the algorithm to query in an N-dimensional space (39). The algorithm uses a majority vote based on ***k*** nearest neighbors to classify which group, or class, the data point belongs to. This classification is compared to the actual label of the data (i.e., the group or class the data point belongs to) and the result is reported as a classification accuracy – or how well the classifier accurately predicted the class, or group, the data belonged to. We applied a leave-one-out approach wherein the algorithm leaves out one data point (i.e., one participant) as a test sample to classify using the remaining data.

**REFERENCES**

1. Makris N, Gasic GP, Kennedy DN, Hodge SM, Kaiser JR, Lee MJ, *et al.* (2008): Cortical Thickness Abnormalities in Cocaine Addiction-A Reflection of Both Drug Use and a Pre-existing Disposition to Drug Abuse? *Neuron* 60: 174–188.

2. Blood AJ, Iosifescu D v., Makris N, Perlis RH, Kennedy DN, Dougherty DD, *et al.* (2010): Microstructural Abnormalities in Subcortical Reward Circuitry of Subjects with Major Depressive Disorder. *PLoS ONE* 5: e13945.

3. Kruskal WH, Wallis WA (1952): Use of Ranks in One-Criterion Variance Analysis. *J Am Stat Assoc* 47: 583–621.

4. Aharon I, Etcoff N, Ariely D, Chabris CF, O’Connor E, Breiter HC (2001): Beautiful faces have variable reward value: fMRI and behavioral evidence. *Neuron* 32: 537–551.

5. Breiter HC, Gasic GP, Makris N (2006): Imaging the Neural Systems for Motivated Behavior and Their Dysfunction in Neuropsychiatric Illness. *Complex Systems Science in Biomedicine* 763–810.

6. Kim BW, Kennedy DN, Lehár J, Lee MJ, Blood AJ, Lee S, *et al.* (2010): Recurrent, robust and scalable patterns underlie human approach and avoidance. *PLoS ONE* 5: e10613.

7. Livengood SL, Sheppard JP, Kim BW, Malthouse EC, Bourne JE, Barlow AE, *et al.* (2017): Keypress-Based Musical Preference Is Both Individual and Lawful. *Frontiers in Neuroscience* 11: 136.

8. Strauss MM, Makris N, Aharon I, Vangel MG, Goodman J, Kennedy DN, *et al.* (2005): fMRI of sensitization to angry faces. https://doi.org/10.1016/j.neuroimage.2005.01.053

9. Viswanathan V, Sheppard JP, Kim BW, Plantz CL, Ying H, Lee MJ, *et al.* (2017): A Quantitative Relationship between Signal Detection in Attention and Approach/Avoidance Behavior. *Frontiers in Psychology* 8: 122.

10. Mellers BA, Schwartz A, Ho K, Ritov I (1997): Decision Affect Theory: Emotional Reactions to the Outcomes of Risky Options. *Psychological Science* 8: 423–429.

11. Mellers B, Schwartz A, Ritpv D (1999): Emotion-Based Choice. *Journal of Experimental Psychology: General* 128: 332–345.

12. Paulus MP (2007): Decision-making dysfunctions in psychiatry - Altered homeostatic processing? *Science (1979)* 318: 602–606.

13. Perlis RH, Holt DJ, Smoller JW, Blood AJ, Lee S, Kim BW, *et al.* (2008): Association of a polymorphism near CREB1 with differential aversion processing in the insula of healthy participants. *Archives of General Psychiatry* 65: 882–892.

14. Gasic GP, Smoller JW, Perlis RH, Sun M, Lee S, Kim BW, *et al.* (2009): BDNF, relative preference, and reward circuitry responses to emotional communication. *American Journal of Medical Genetics, Part B: Neuropsychiatric Genetics* 150: 762–781.

15. Viswanathan V, Lee S, Gilman JM, Kim BW, Lee N, Chamberlain L, *et al.* (2015): Age-related striatal BOLD changes without changes in behavioral loss aversion. *Frontiers in Human Neuroscience* 9: 176.

16. Bechara A (2005): Decision making, impulse control and loss of willpower to resist drugs: a neurocognitive perspective. *Nature Neuroscience 2005 8:11* 8: 1458–1463.

17. Goldstein RZ, Tomasi D, Alia-Klein N, Cottone LA, Zhang L, Telang F, Volkow ND (2007): Subjective sensitivity to monetary gradients is associated with frontolimbic activation to reward in cocaine abusers. *Drug and Alcohol Dependence* 87: 233–240.

18. Kalivas PW, Volkow ND (2005): The neural basis of addiction: a pathology of motivation and choice. *Am J Psychiatry* 162: 1403–1413.

19. Forbes EE, Hariri AR, Martin SL, Silk JS, Moyles DL, Fisher PM, *et al.* (2009): Altered striatal activation predicting real-world positive affect in adolescent major depressive disorder. *American Journal of Psychiatry* 166: 64–73.

20. Liu L, Zeng LL, Li Y, Ma Q, Li B, Shen H, Hu D (2012): Altered Cerebellar Functional Connectivity with Intrinsic Connectivity Networks in Adults with Major Depressive Disorder. *PLOS ONE* 7: e39516.

21. Manelis A, Almeida JRC, Stiffler R, Lockovich JC, Aslam HA, Phillips ML (2016): Anticipation-related brain connectivity in bipolar and unipolar depression: a graph theory approach. *Brain* 139: 2554–2566.

22. Smoski MJ, Lynch TR, Rosenthal MZ, Cheavens JS, Chapman AL, Krishnan RR (2008): Decision-making and risk aversion among depressive adults. *Journal of Behavior Therapy and Experimental Psychiatry* 39: 567–576.

23. Pedersen ML, Ironside M, Amemori KI, McGrath CL, Kang MS, Graybiel AM, *et al.* (2021): Computational phenotyping of brain-behavior dynamics underlying approach-avoidance conflict in major depressive disorder. *PLOS Computational Biology* 17: e1008955.

24. Ironside M, Amemori K ichi, McGrath CL, Pedersen ML, Kang MS, Amemori S, *et al.* (2020): Approach-Avoidance Conflict in Major Depressive Disorder: Congruent Neural Findings in Humans and Nonhuman Primates. *Biol Psychiatry* 87: 399–408.

25. Filipek PA, Richelme C, Kennedy DN, Caviness VS (1994): The young adult human brain: an MRI-based morphometric analysis. *Cereb Cortex* 4: 344–360.

26. Caviness VS, Meyer J, Makris N, Kennedy DN (1996): MRI-Based Topographic Parcellation of Human Neocortex: An Anatomically Specified Method with Estimate of Reliability. *J Cogn Neurosci* 8: 566–587.

27. Caviness VS, Kennedy DN, Richelme C, Rademacher J, Filipek PA (1996): The human brain age 7-11 years: a volumetric analysis based on magnetic resonance images. *Cereb Cortex* 6: 726–736.

28. Makris N, Gasic GP, Seidman LJ, Goldstein JM, Gastfriend DR, Elman I, *et al.* (2004): Decreased absolute amygdala volume in cocaine addicts. *Neuron* 44: 729–740.

29. Makris N, Kaiser J, Haselgrove C, Seidman LJ, Biederman J, Boriel D, *et al.* (2006): Human cerebral cortex: A system for the integration of volume-and surface-based representations. https://doi.org/10.1016/j.neuroimage.2006.04.220

30. Worth AJ, Makris N, Caviness VS, Kennedy DN (1997): Neuroanatomical Segmentation in MRI: Technological Objectives. *International Journal of Pattern Recognition and Artificial Intelligence* 11: 1161–1187.

31. Seidman LJ, Faraone S v., Goldstein JM, Goodman JM, Kremen WS, Toomey R, *et al.* (1999): Thalamic and amygdala–hippocampal volume reductions in first-degree relatives of patients with schizophrenia: an MRI-based morphometric analysis. *Biological Psychiatry* 46: 941–954.

32. Seidman LJ, Faraone S v., Goldstein JM, Kremen WS, Horton NJ, Makris N, *et al.* (2002): Left Hippocampal Volume as a Vulnerability Indicator for Schizophrenia: A Magnetic Resonance Imaging Morphometric Study of Nonpsychotic First-Degree Relatives. *Archives of General Psychiatry* 59: 839–849.

33. Goldstein JM, Goodman JM, Seidman LJ, Kennedy DN, Makris N, Lee H, *et al.* (1999): Cortical Abnormalities in Schizophrenia Identified by Structural Magnetic Resonance Imaging. *Arch Gen Psychiatry* 56: 537–547.

34. Breiter HC, Filipek PA, Kennedy DN, Baer L, Pitcher DA, Olivares MJ, *et al.* (1994): Retrocallosal White Matter Abnormalities in Patients With Obsessive-compulsive Disorder. *Archives of General Psychiatry* 51: 663–664.

35. Fischl B, Salat DH, Busa E, Albert M, Dieterich M, Haselgrove C, *et al.* (2002): Whole brain segmentation: automated labeling of neuroanatomical structures in the human brain. *Neuron* 33: 341–355.

36. Fischl B, Salat DH, van der Kouwe AJW, Makris N, Ségonne F, Quinn BT, Dale AM (2004): Sequence-independent segmentation of magnetic resonance images. *Neuroimage* 23 Suppl 1. https://doi.org/10.1016/J.NEUROIMAGE.2004.07.016

37. Kahneman D, Tversky A (1979): On the interpretation of intuitive probability: A reply to Jonathan Cohen. *Cognition* 7: 409–411.

38. Markowitz H (1952): The utility of wealth. *Journal of Political Economy* 60: 151–158.

39. Keller JM, Gray MR (1985): A Fuzzy K-Nearest Neighbor Algorithm. *IEEE Transactions on Systems, Man and Cybernetics* SMC-15: 580–585.
