# Supplementary material for: Characterizing major depressive disorder and substance use disorder using heatmaps and variable interactions: The utility of operant behavior and brain structure relationships
Source: PLoS One. 2024 Mar 11;19(3):e0299528. doi: 10.1371/journal.pone.0299528 (PMC10927130; doi:10.1371/journal.pone.0299528)
Supplement: S1 Fig — Results were plotted using regression results from Table 1 where covariates were included. (DOCX) [file pone.0299528.s004.docx]

**S1 Figure**. Structure-behavior regression plots. (A) CTRL plots, (B) MDD plots, (C) CD plots. Note that covariates were not included when plotting these relationships.
