# Supplementary material for: Characterizing major depressive disorder and substance use disorder using heatmaps and variable interactions: The utility of operant behavior and brain structure relationships
Source: PLoS One. 2024 Mar 11;19(3):e0299528. doi: 10.1371/journal.pone.0299528 (PMC10927130; doi:10.1371/journal.pone.0299528)
Supplement: S2 Fig — (DOCX) [file pone.0299528.s005.docx]

**S2 Figure**. Overlapping structure-behavior regressions without covariate inclusion. (A) Common regressions between CTRL and MDD. (B) Common regressions between CTRL and CD. (C) Common regressions between MDD and CD. (D) Summary of all common regressions between the three groups; beta agreeance indicates if the beta terms (slopes) had the same directionality (+/+ or -/-) or not (+/-).
