# Supplementary material for: Characterizing major depressive disorder and substance use disorder using heatmaps and variable interactions: The utility of operant behavior and brain structure relationships
Source: PLoS One. 2024 Mar 11;19(3):e0299528. doi: 10.1371/journal.pone.0299528 (PMC10927130; doi:10.1371/journal.pone.0299528)
Supplement: S1 Table — (DOCX) [file pone.0299528.s006.docx]

**S1 Table**. Demographic Summaries. (A) CTRL = control, MDD = major depressive disorder, CD = cocaine dependence; gender (male, female); handedness (right, left); race (White, Black, Asian). (B) Age and years of education for each group. (C) Demographic and drug-use data for the entire cohort. Mean and standard deviation data listed under the first Addicts column includes money in dollars, and days of use over the prior 30 days before the study. The column with “# non-zero” represents the number of subjects with non-zero responses.
