# Supplementary material for: Characterizing major depressive disorder and substance use disorder using heatmaps and variable interactions: The utility of operant behavior and brain structure relationships
Source: PLoS One. 2024 Mar 11;19(3):e0299528. doi: 10.1371/journal.pone.0299528 (PMC10927130; doi:10.1371/journal.pone.0299528)
Supplement: S4 Table — (DOCX) [file pone.0299528.s009.docx]

**S4 Table**. Brain volume differences by demographic variables. Brain volume abbreviations are defined in Supplemental Table 3.
