# Supplementary material for: Characterizing major depressive disorder and substance use disorder using heatmaps and variable interactions: The utility of operant behavior and brain structure relationships
Source: PLoS One. 2024 Mar 11;19(3):e0299528. doi: 10.1371/journal.pone.0299528 (PMC10927130; doi:10.1371/journal.pone.0299528)
Supplement: S5 Table — Overlapping regressions presented in Fig 2 with inclusion of covariates were excluded. (DOCX) [file pone.0299528.s010.docx]

**S5 Table.** Overlapping brain regions using structure-behavior regression relationships (with covariates) presented in Table 1 (excluding overlapping regressions presented in Fig 2 with inclusion of covariates).
