# Supplementary figures and images for: Characterizing major depressive disorder and substance use disorder using heatmaps and variable interactions: The utility of operant behavior and brain structure relationships
Source: PLoS One. 2024 Mar 11;19(3):e0299528. doi: 10.1371/journal.pone.0299528 (PMC10927130; doi:10.1371/journal.pone.0299528)

**S6 Table**. Structure-behavior regressions without covariate inclusion. (A) CTRL, (B) MDD, (C) CD.


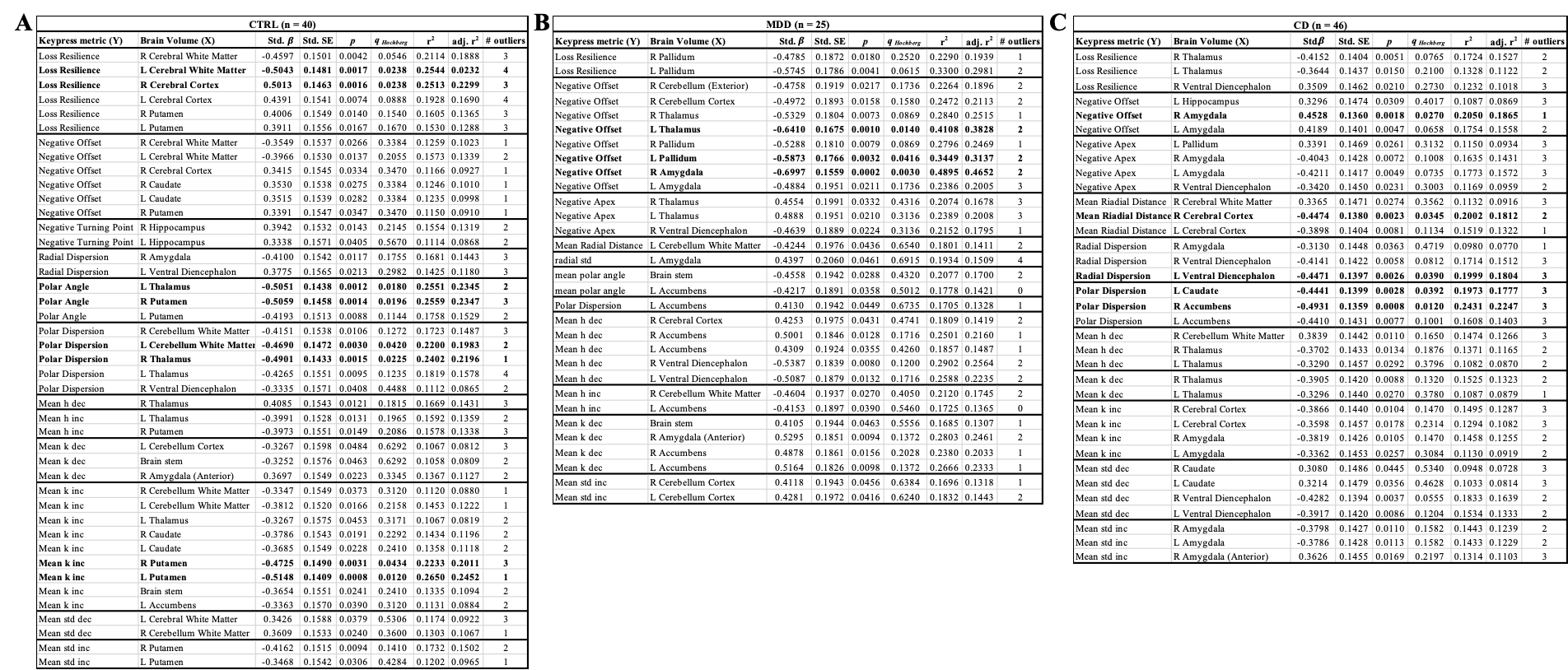

Supplement: S6 Table — (DOCX) [file pone.0299528.s011.docx]
