# Supplementary material for: Characterizing major depressive disorder and substance use disorder using heatmaps and variable interactions: The utility of operant behavior and brain structure relationships
Source: PLoS One. 2024 Mar 11;19(3):e0299528. doi: 10.1371/journal.pone.0299528 (PMC10927130; doi:10.1371/journal.pone.0299528)
Supplement: S7 Table — Overlapping regressions presented in S2 Fig without inclusion of covariates were excluded. (DOCX) [file pone.0299528.s012.docx]

**S7 Table.** Overlapping brain regions using structure-behavior regression relationships (without covariates) presented in S6 Table (excluding overlapping regressions presented in S2 Fig without inclusion of covariates).
